# Impact of a Serious Game (Escape COVID-19) on the Intention to Change COVID-19 Control Practices Among Employees of Long-term Care Facilities: Web-Based Randomized Controlled Trial

**DOI:** 10.2196/27443

**Published:** 2021-03-25

**Authors:** Mélanie Suppan, Mohamed Abbas, Gaud Catho, Loric Stuby, Simon Regard, Sophia Achab, Stephan Harbarth, Laurent Suppan

**Affiliations:** 1 Division of Anesthesiology Department of Anesthesiology, Clinical Pharmacology, Intensive Care and Emergency Medicine University of Geneva Hospitals and Faculty of Medicine Geneva Switzerland; 2 Infection Control Programme and WHO Collaborating Centre on Patient Safety University of Geneva Hospitals and Faculty of Medicine Geneva Switzerland; 3 Genève TEAM Ambulances Geneva Switzerland; 4 Division of Emergency Medicine Department of Anesthesiology, Clinical Pharmacology, Intensive Care and Emergency Medicine University of Geneva Hospitals and Faculty of Medicine Geneva Switzerland; 5 Division of the Surgeon General Geneva Directorate of Health Geneva Switzerland; 6 Specialized Facility in Behavioral Addictions ReConnecte University of Geneva Hospitals Geneva Switzerland; 7 WHO Collaborating Center in Training and Research in Mental Health Faculty of Medicine University of Geneva Geneva Switzerland

**Keywords:** COVID-19, transmission, serious game, infection prevention, health care worker, SARS-CoV-2, nursing homes, randomized controlled trial, long-term care facilities, impact, game, intention, control, elderly

## Abstract

**Background:**

Most residents of long-term care facilities (LTCFs) are at high risk of complications and death following SARS-CoV-2 infection. In these facilities, viral transmission can be facilitated by shortages of human and material resources, which can lead to suboptimal application of infection prevention and control (IPC) procedures. To improve the dissemination of COVID-19 IPC guidelines, we developed a serious game called “Escape COVID-19” using Nicholson’s RECIPE for meaningful gamification, as engaging serious games have the potential to induce behavioral change.

**Objective:**

As the probability of executing an action is strongly linked to the intention of performing it, the objective of this study was to determine whether LTCF employees were willing to change their IPC practices after playing “Escape COVID-19.”

**Methods:**

This was a web-based, triple-blind, randomized controlled trial, which took place between November 5 and December 4, 2020. The health authorities of Geneva, Switzerland, asked the managers of all LTCFs under their jurisdiction to forward information regarding the study to all their employees, regardless of professional status. Participants were unaware that they would be randomly allocated to one of two different study paths upon registration. In the control group, participants filled in a first questionnaire designed to gather demographic data and assess baseline knowledge before accessing regular online IPC guidelines. They then answered a second questionnaire, which assessed their willingness to change their IPC practices and identified the reasons underlying their decision. They were then granted access to the serious game. Conversely, the serious game group played “Escape COVID-19” after answering the first questionnaire but before answering the second one. This group accessed the control material after answering the second set of questions. There was no time limit. The primary outcome was the proportion of LTCF employees willing to change their IPC practices. Secondary outcomes included the factors underlying participants’ decisions, the domains these changes would affect, changes in the use of protective equipment items, and attrition at each stage of the study.

**Results:**

A total of 295 answer sets were analyzed. Willingness to change behavior was higher in the serious game group (82% [119/145] versus 56% [84/150]; *P*<.001), with an odds ratio of 3.86 (95% CI 2.18-6.81; *P*<.001) after adjusting for professional category and baseline knowledge, using a mixed effects logistic regression model with LTCF as a random effect. For more than two-thirds (142/203) of the participants, the feeling of playing an important role against the epidemic was the most important factor explaining their willingness to change behavior. Most of the participants unwilling to change their behavior answered that they were already applying all the guidelines.

**Conclusions:**

The serious game “Escape COVID-19” was more successful than standard IPC material in convincing LTCF employees to adopt COVID-19–safe IPC behavior.

**International Registered Report Identifier (IRRID):**

RR2-10.2196/25595

## Introduction

### Background and Importance

Long-term care facilities (LTCFs) have been hit hard by the COVID-19 pandemic [1-4]. Although most LTCF residents are either old or frail and therefore more prone to complications and death following SARS-CoV-2 infection [5,6], other factors, such as shortages of human resources [7-9], must also be taken into account. This lack of resources can lead to suboptimal application of infection prevention and control (IPC) procedures [10], thus facilitating viral transmission. As even a single LTCF employee can contaminate a great number of colleagues and residents [11], and as rapid viral transmission between residents has been reported [12], it is essential to support LTCF employees [13] and ultimately help them adhere to IPC guidelines [14].

Even though most LTCF employees recognize the importance of preventing SARS-CoV-2 transmission, their motivation to act according to their awareness might be hampered by overwork [8,15], by too often updated and sometimes contradicting guidelines [16,17], and by mistrust in health care authorities [18]. Moreover, in spite of well-established evidence regarding specific IPC practices such as hand hygiene and use of personal protective equipment (PPE) [19], most health care workers (HCWs), including LTCF employees, only seldom apply them perfectly [20-22]. The current need for physical distance and the disruption of the regular continuing medical education courses has deteriorated this situation even further [23].

To enhance the communication of appropriate IPC guidelines and improve their application by HCWs and related staff who are regularly in contact with patients, we developed a web-based serious game called “Escape COVID-19” [24] using Nicholson’s RECIPE for meaningful gamification [25]. Although electronic learning (e-learning) interventions might be ineffective in teaching complex technical procedures ([17,26]; Stuby et al, unpublished data, 2021), they are nevertheless useful in increasing knowledge and their use is generally associated with a high level of learner satisfaction [27-29]. As the probability of executing an action is strongly linked to the intention of performing it (according to the theory of planned behavior [30,31]), an engaging serious game could enhance the dissemination of essential IPC guidelines and encourage LTCF employees to change their behavior regarding IPC practices [29,32,33].

### Objective

Our main objective was to assess the impact of “Escape COVID-19,” a web-based serious game, on the intention of LTCF employees to change their IPC practices. Our secondary objective was to determine the reasons underlying the potential willingness to change IPC practices or lack thereof.

## Methods

### Study Design and Setting

**Figure 1:**
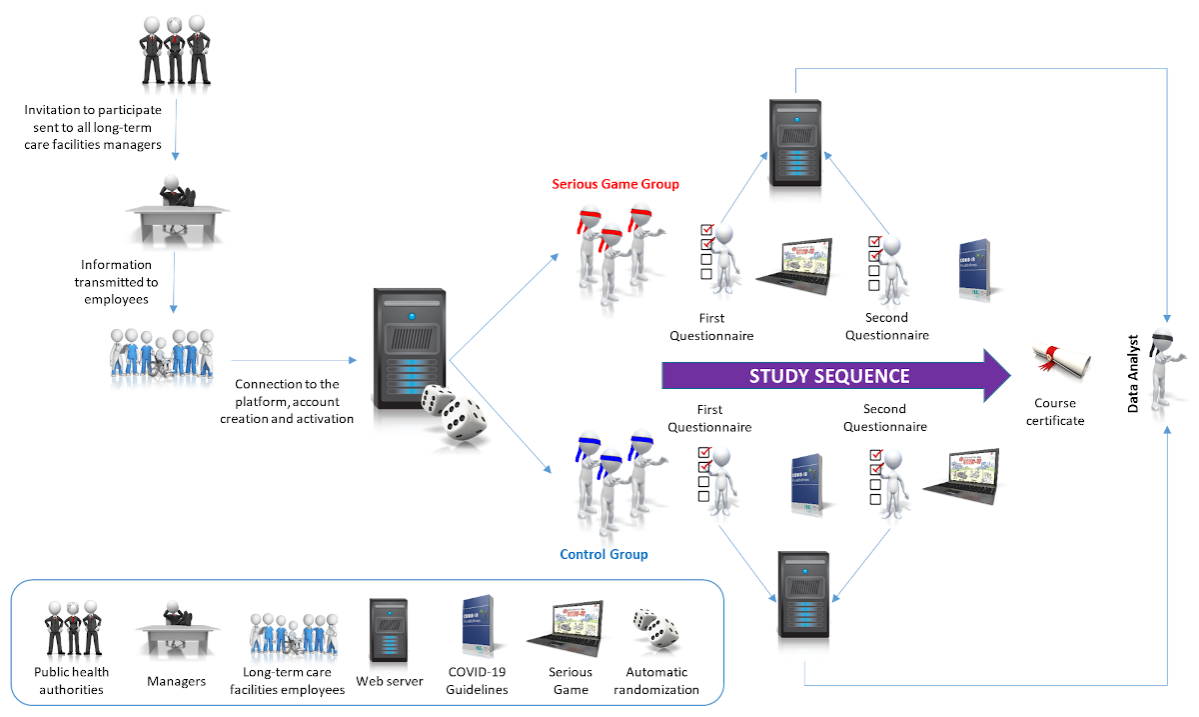
Study design.

This was a web-based, triple-blind (investigator, participants, data analyst), randomized controlled trial, which took place between November 5 and December 4, 2020. Its design has been published previously [34] and is summarized in Figure 1. A declaration of no objection was issued by our regional ethics committee (Req-2020-01262) as such projects do not fall within the scope of the Swiss federal law on human research [35]. This study was designed according to the CONSORT-EHEALTH (Consolidated Standards of Reporting Trials) guidelines [36] and incorporates relevant elements from the CHERRIES (Checklist for Reporting Results of Internet E-Surveys) checklist [37].

Having been informed by IPC specialists from the Geneva University Hospitals of the development of “Escape COVID-19,” the public health authorities of Geneva, Switzerland, were interested in giving the employees of all LTCFs under their jurisdiction access to this serious game. They therefore provided us with a comprehensive list of all such facilities, but insisted on liaising with LTCF managers themselves. As LTCF employees other than HCWs can often be in contact with residents, we informed the representatives of the health care authorities that, for methodological and clinical relevance, all LTCF employees should be invited to participate, regardless of their professional status. We therefore provided them with an email template that described the objective of this study and the target population, and gave information regarding data protection (Multimedia Appendix 1). This template was sent by the public health authorities to all LTCF managers following a regular LTCF coordination meeting during which the characteristics of the study and the serious game were detailed. Participants, including LTCF managers, were unaware that they would be allocated to one of two different study arms but were required to provide consent (see below) and were given the approximate time required to complete the whole path (30 minutes). An email address that could be used to contact the investigators was also provided. The only incentive that participants were given was the promise of acquiring a course completion certificate at the end of the study path.

### Online Platform

**Figure 2:**
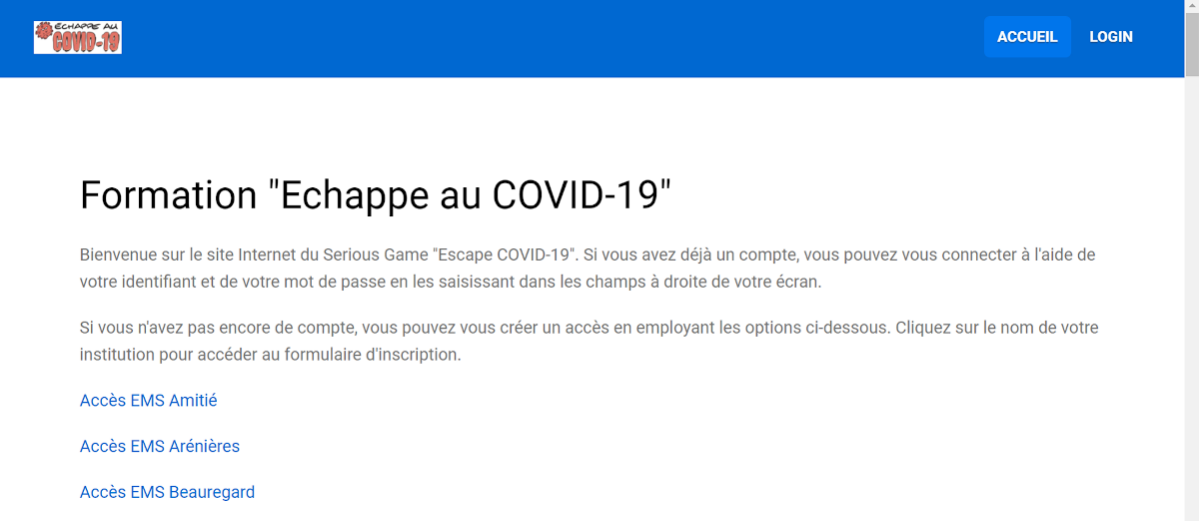
Front page of the study website (in French). Only the first three long-term care facilities are displayed.

A specific web-based platform [38], hosted on a Swiss server, was created under the Joomla 3.9 content management system [39]. Only two authors (MS and L Suppan) had access to the administration console and data, which were stored on an encrypted MySQL-compatible database (MariaDB 5.5.5, MariaDB Foundation). Daily backups were performed throughout the study period. They were triggered by a cron job script and uploaded on a physically separate server through an encrypted connection. The front page displayed a comprehensive list of all LTCFs under Geneva health authorities’ jurisdiction (Figure 2).

Stratification was achieved by having LTCF employees click on a link specific to their institution. When they clicked on this link, participants were automatically randomized to one of the two study paths by GegaByte’s Random Article module (GegaByte Technologies) [40]. This process was invisible to the end user and there was no way either participants or investigators could influence group allocation.

**Figure 3:**
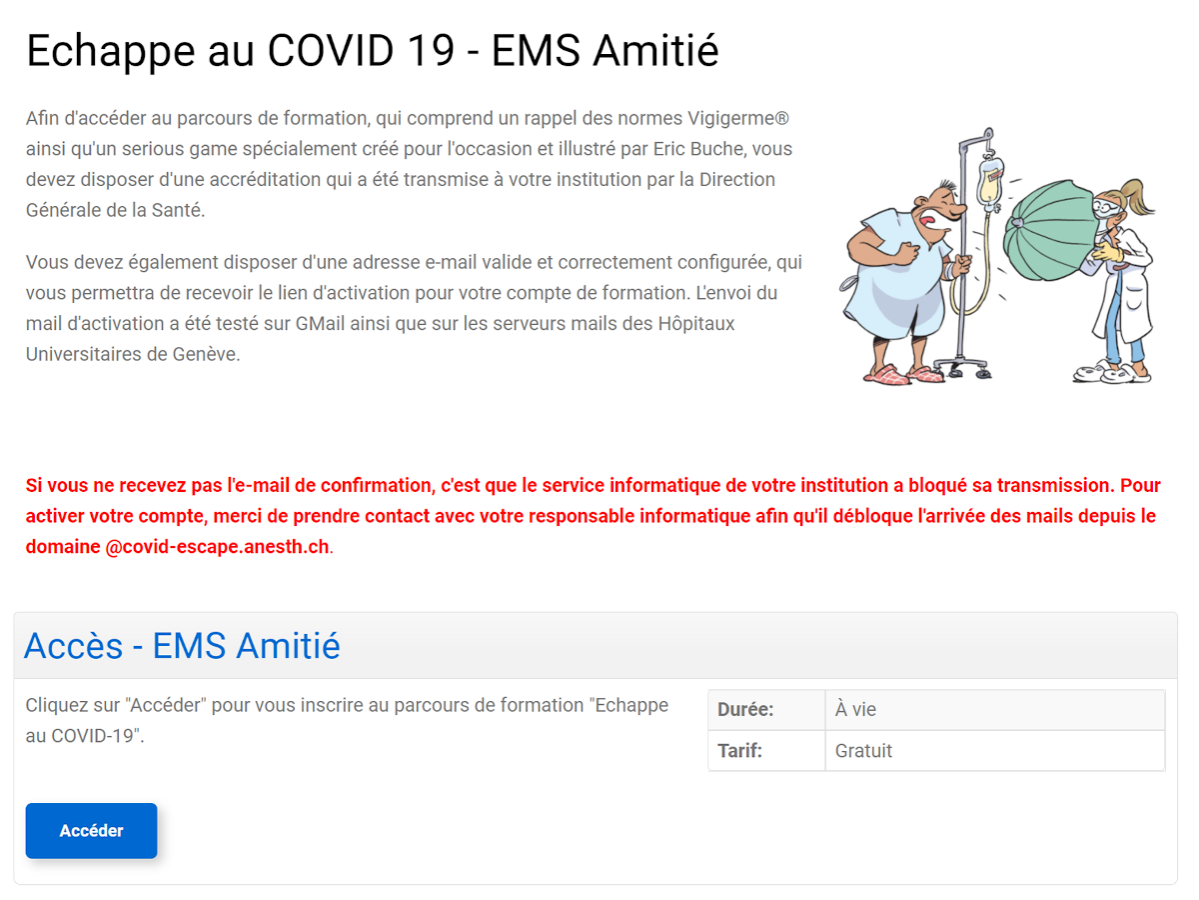
General information displayed (in French) before accessing the registration form.

General information regarding the study, the need to register with a valid email address, and the need to allow the reception of emails coming from the website’s domain was then displayed (Figure 3).

**Figure 4:**
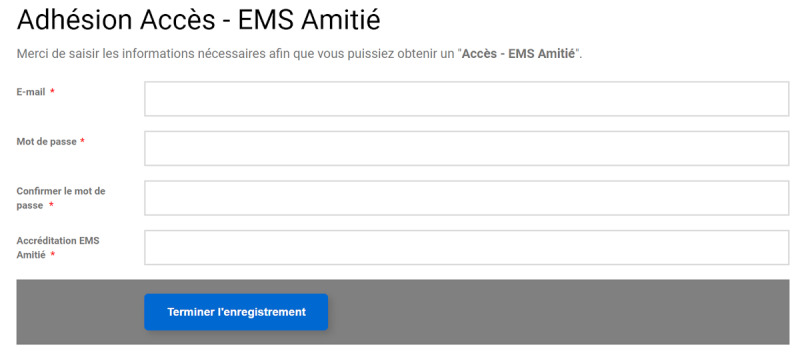
Registration form (in French).

After clicking on the “Access” button, a registration form, identical for both groups, was shown (Figure 4). This form was created using Membership Pro (Joomdonation) as this component enabled us to allocate users to specific groups, to create specific fields, and to disable the “Name” field [41]. Therefore, participants were only asked to enter their email address; they were not asked for any other personal information and were not required to give their first and last names.

To avoid having LTCF employees registering under the wrong institution, LTCF-specific accreditations were created for each facility (Figure 4, “Accréditation” field). The full list of accreditations was sent to the public health authorities, who were informed they were to adapt our email template accordingly for each LTCF.

After filling in the registration form, participants were asked to activate their account using a specific link sent to the email address they had entered. This email also contained a reminder regarding data handling and security (Multimedia Appendix 2). Participants were informed that clicking on the activation link was considered as consent to participate in the study. After activating their account, participants were able to log in to the platform. Upon login, they were redirected to the first questionnaire (Multimedia Appendix 3) by the Redirect-on-Login 4.6 component (Pages-and-Items) [42].

This first questionnaire (Multimedia Appendix 3) was designed to assess the participants’ baseline knowledge and to gather demographic data. It was developed using Community Surveys Pro (Corejoomla) [43], which enables a completeness check and allows for the use of branching logic. The number of initial questions was kept to a minimum and branching logic was used to try to limit attrition [44-47]. All multiple-choice and multiple-answer questions were mandatory and had to be answered before participants could move on to the next step. The answers could not be changed once a page had been completed.

**Figure 5:**
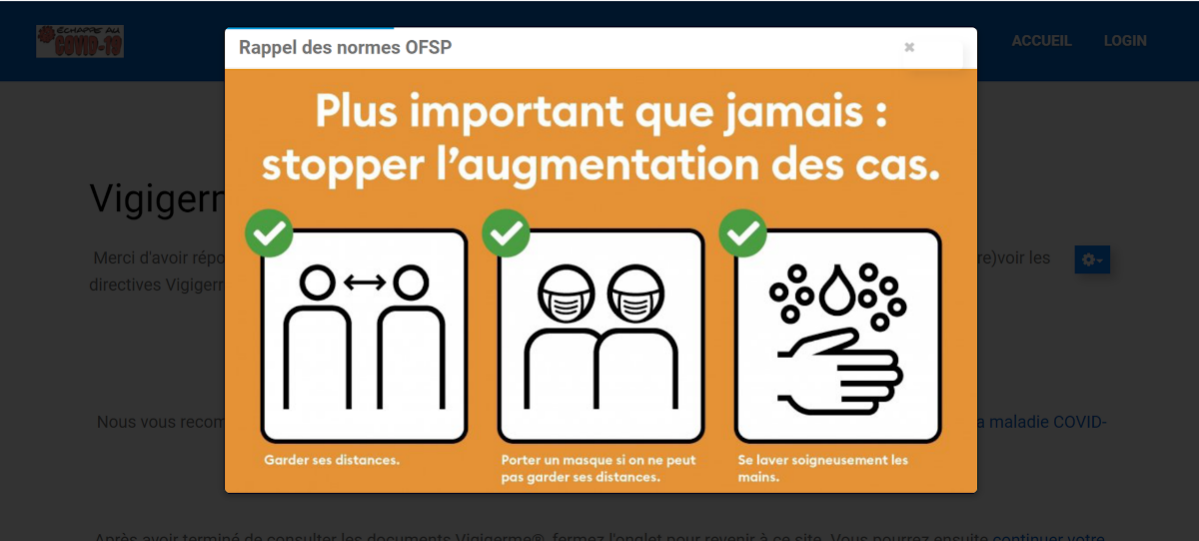
Control material (in French). The model was displayed for 10 seconds before providing links to infection prevention and control guidelines.

After completing this first questionnaire, participants in the control group were shown a quick reminder of the most recent national guidelines published by the Federal Office of Public Health of the Swiss Confederation [48]. They were also given links to local IPC guidelines for health care professionals (Vigigerme) provided by the Geneva University Hospitals (Figure 5). After reviewing these guidelines, which are freely available on the internet [49], these participants were asked to complete the second questionnaire (Multimedia Appendix 4).

**Figure 6:**
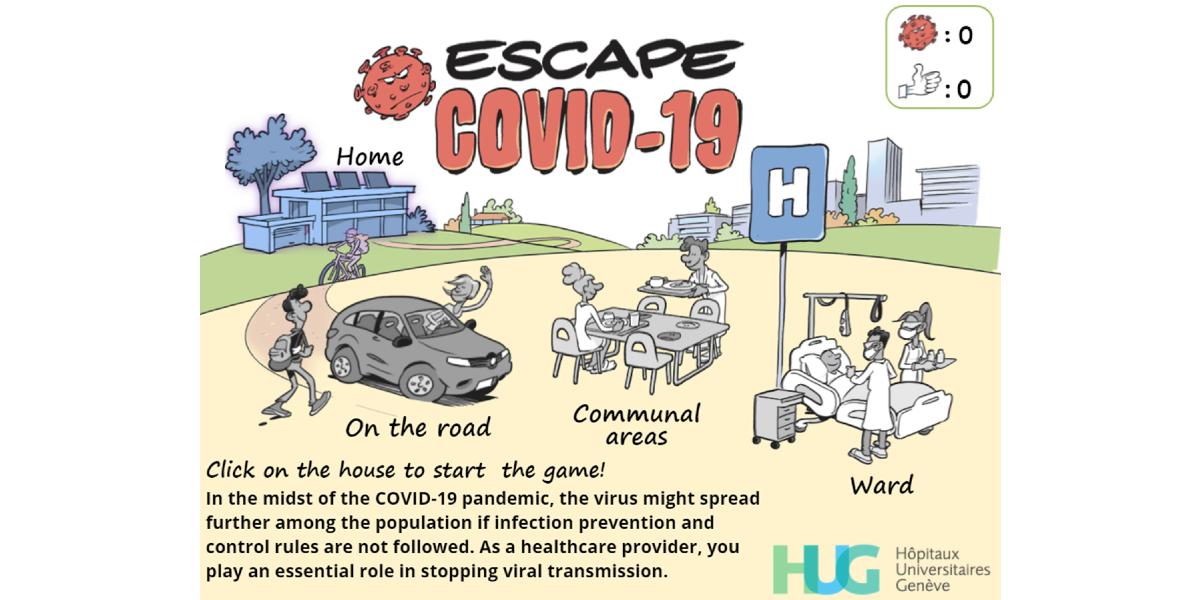
Welcome screen of the Escape COVID-19 serious game.

**Figure 7:**
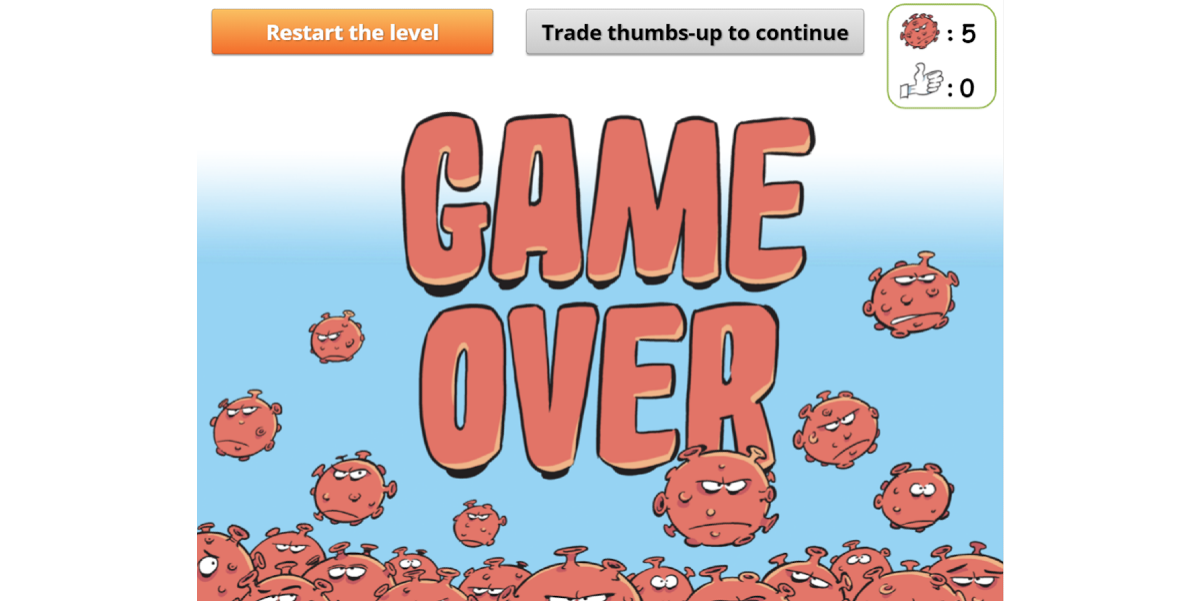
"Game over" screen of the Escape COVID-19 serious game.

Conversely, “Escape COVID-19” (version 2.1.1) was launched once participants in the serious game group had completed the first questionnaire. The development of this serious game has previously been described [24]. Briefly, “Escape COVID-19” was first developed in French under Articulate Storyline 3 (Articulate Global) through multiple iterations by using the first three steps of the SERES framework [23,50,51] and Nicholson’s RECIPE for meaningful gamification [25]. Graphical elements were designed by Eric Buche [52] to lend a unique aspect to the game (Figures 6 and 7). The game has been fully translated in English and can be freely accessed on the internet [53]. The first and last author can be contacted to obtain Shareable Content Object Reference Model (SCORM) packages, which can be reused freely for research and educational purposes. Although the screen captures are presented in English to enhance their comprehension by an international readership, the original (French) versions can be seen in Multimedia Appendix 5.

This serious game includes four different levels, which create a meaningful narrative [25] by presenting recurrent and critical standard situations encountered by HCWs, including community and hospital exposures. Feedback is systematically given [54] to reinforce the expected behavior. Desirable behaviors and correct answers are rewarded by awarding a “thumbs-up,” while viruses are accumulated whenever a wrong answer is given or an unwished-for behavior is performed. A “game over” screen is displayed (Figure 7) if the player gathers a total of 5 viruses. The player can then either restart the level or spend their thumbs-up to decrease the virus count (1:1).

**Figure 8:**
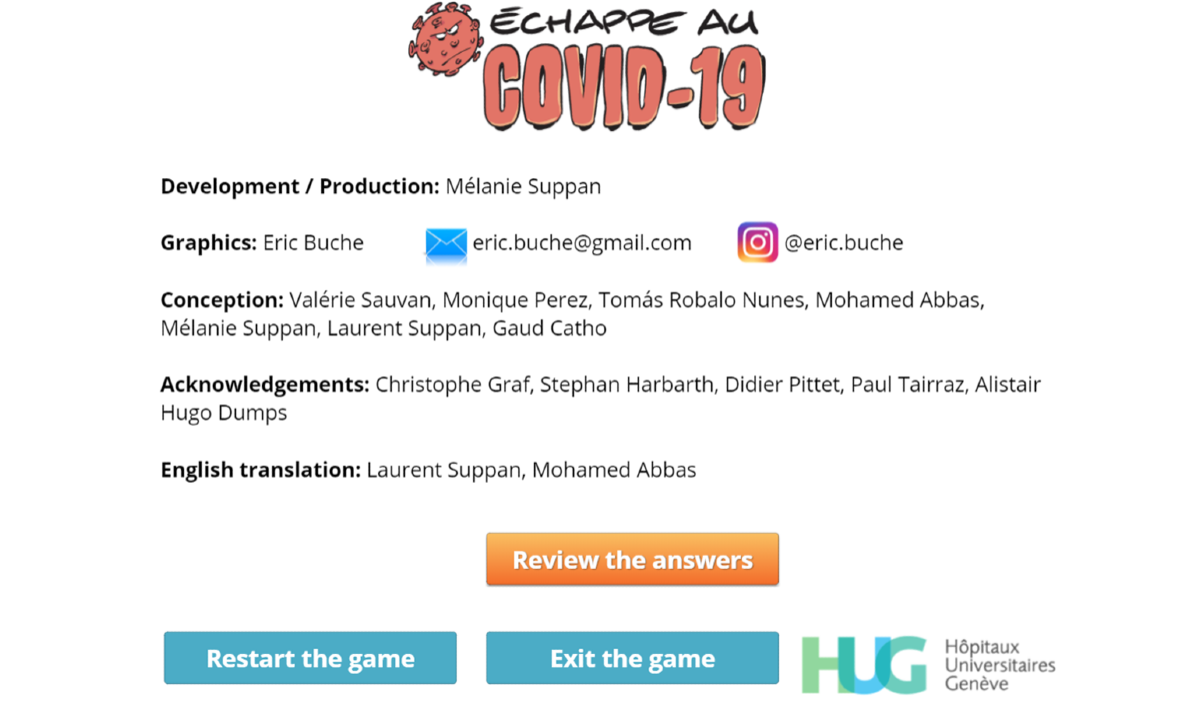
The only way participants could access the second questionnaire was by clicking on the “Exit the game” button.

After completing all four levels, participants were shown a link that enabled them to access the second questionnaire (Figure 8). This ensured that this questionnaire could only be answered by participants who had actually completed the game.

This second and last questionnaire was designed to assess whether the participants intended to change their IPC practices after completing their allocated set of learning materials. It was also developed using Community Surveys Pro, with branching logic used to try to limit attrition and completeness checks enabled to avoid missing data. After completing this last questionnaire, participants in the control group were granted access to the serious game, while those who belonged to the serious game group were shown the control materials. All participants could then access a certificate generation module that enabled them to obtain a course completion certificate. No time limit was set for either of the two questionnaires or for completing the serious game.

During the whole study path, participants were allocated to specific user groups according to their progression. This was carried out by using Joomla’s native Access Control List feature (PHP functions JUserHelper::addUserToGroup and JUserHelper::removeUserFromGroup, embedded using Sourcerer by Regular Labs [55]), and served two purposes. First, it allowed us to determine precisely at which step some participants elected to abandon the study. Second, it allowed participants who had been interrupted to be immediately redirected to the next incomplete step of the study when resuming.

### Outcomes

The primary outcome was the proportion of LTCF employees who answered they were willing to change their IPC practices after seeing either the serious game or the control material. The secondary outcomes were the identification of factors associated with participant willingness to change their behavior, the reasons given by participants opposed to changing theirs [56], and the potential motivators which could have led them to change. Attrition was evaluated at each stage of the study.

We also assessed the IPC domains affected in participants who answered they were willing to change their IPC behavior, and whether these participants would modify their use of specific PPE items. In total, 13 questions were used to assess these latter outcomes. Therefore, conversely to the previous outcomes, answering questions related to IPC domains and PPE items were not compulsory to limit attrition.

### Participants and Sample Size

Regardless of their professional status, all LTCF employees working in Geneva, Switzerland, who received the invitation and elected to participate were included in this study and represented a convenience sample. As the number of eligible employees was estimated to be approximately 4000 people, we hoped for a participation rate of around 20%. This would have allowed us to detect a difference of 10% at the .05 significance level with a power of 80% as we had calculated that 388 participants would have been needed in each group to detect such a difference.

Answer sets that had not been marked as completed by the computer system and those filled by participants other than LTCF employees (ie, government employees or employees of other institutions) were excluded.

### Data Curation and Statistical Analysis

Data was exported by L Suppan in Microsoft Excel (Microsoft Corp; XLSX) and in comma-separated value (CSV) formats depending on the components before being imported, appended, and merged under Stata (StataCorp LLC). The groups were renamed using neutral names (“Atreides” and “Corrino”), the fields that could have led to the unblinding of the data analyst were removed, and the curated DTA file was transmitted to L Stuby, who used Stata (version 15.1) for statistical analysis. This investigator was not part of the serious game development team and did not coauthor the original publication, as we wanted to avoid any potential conflict of interest.

Univariable and multivariable logistic regression were used to assess the primary outcome, with adjustment performed according to prior knowledge (expressed as the percentage of correct answers), professional status, and facility. The log-linearity assumption was checked graphically and the goodness-of-fit was tested using the Hosmer-Lemeshow test. As randomization was stratified by center, we adjusted for this in the analysis by employing a random effects logistic regression model, using LTCF as a random effect. We tested the null hypothesis of absence of random effect using a chi-bar-square test. We calculated the intraclass correlation coefficient (ICC) to quantify to what extent responses in a single LTCF were correlated.

Secondary outcomes were analyzed by assigning numerical values to the answers gathered through the use of Likert scales. As the domains potentially affected by a change in behavior were assessed using Likert scales ranging from 1 (not at all) to 6 (very much), the same values (ie, a score ranging from 1 to 6) were assigned to each item. The composite outcome was the sum of these 9 questions and was analyzed through univariable linear regression then adjusted by employing a mixed effects model, using LTCF as a random effect and the same adjustment variables as for the primary outcome.

The changes in the use of specific PPE items were assessed using 5-point Likert scales, ranging from “much less” to “much more.” An odd number was decided upon to allow participants to give a neutral answer. Values ranging from –2 to +2 were therefore assigned to each answer, with positive values attributed to changes enhancing IPC behavior. A composite outcome was generated by summing up these values. We used the same statistical method as for the prior composite outcome. When computing composite outcomes, the same weight was applied to all questions. As a reduction in the use of N95 respirators can also be considered as desirable depending on the setting, a sensitivity analysis was performed by analyzing the composite outcome with and without this particular item. Each individual question based on a Likert scale was also analyzed separately and the results are presented graphically, either in the manuscript or in multimedia appendices. No imputation technique was used.

Descriptive statistics were used to describe the factors associated with willingness or refusal to change behavior. The chi-square test or—if the expected frequencies assumption was not met—the Fisher exact test were used to assess differences between groups.

The curated data file (with the groups renamed as “Control” and “Serious Game”) is available on the Mendeley Data repository [57].

## Results

**Figure 9:**
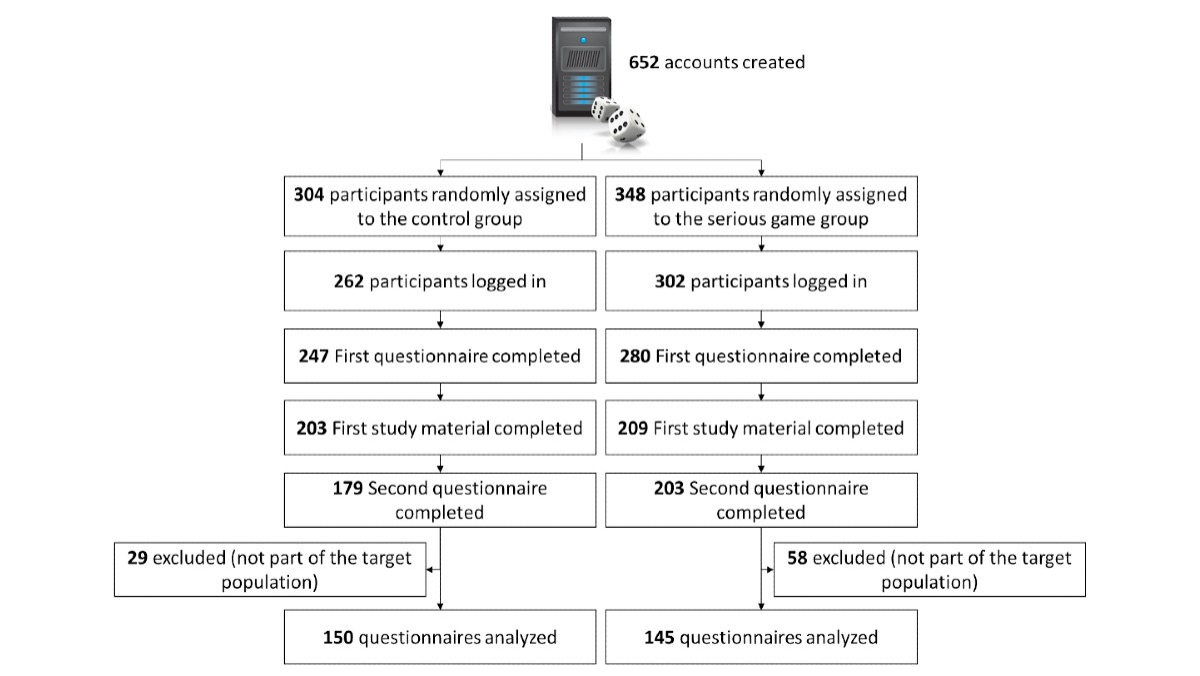
Study flowchart.

During the course of the study, the public health authorities of Geneva requested that we create specific accesses for other institutions whose members were not part of the target population. A total of 652 accounts were created, out of which 569 (87.3%) were activated. After exclusion of accounts that had not completed the second questionnaire and of those belonging to institutions other than LTCFs, 295 answer sets were analyzed (Figure 9). Participant characteristics are detailed in Table 1.

**Table 1:** Participant characteristics from 36 long-term care facilities in Geneva, Switzerland. Totals may not equal 100% due to rounding.

Participant characteristics		Control (n=150)	Serious game (n=145)
Gender, female, n (%)		118 (78.7)	111 (76.6)
Age (years), median (Q1-Q3)		45 (39-51)	43 (34-52)
**Professional group, n (%)**			
	Nursing staff	83 (55.3)	83 (57.2)
	Administrative/support staff	30 (20.0)	24 (16.6)
	Physicians	3 (2.0)	3 (2.1)
	Other	34 (22.7)	35 (24.1)
**Detailed nursing staff status, n (%)**			
	Nurse	32 (38.6)	35 (41.2)
	Health care assistant	36 (43.4)	34 (40.0)
	Nurse assistant	5 (6.0)	11 (12.9)
	Other	10 (12.1)	5 (5.9)
**Non–health care professionals, n (%)**			
	Hospitality/catering	20 (58.8)	14 (40.0)
	Animation	11 (32.4)	12 (34.3)
	Other	3 (8.8)	9 (25.7)
**Self-assessed frequency of patient contact for non–health care professionals, n (%)**			
	Very often	25 (39.1)	25 (41.0)
	Quite often	19 (29.7)	12 (19.7)
	Infrequently	15 (23.4)	22 (36.1)
	Almost never	5 (7.8)	2 (3.3)
Years active in the health sector, median (Q1-Q3)		11 (6-22)	12 (4-21)
Baseline knowledge (percentage of correct answers), median (Q1-Q3)		40 (20-60)	40 (20-80)

Out of 36 LTCFs, only 8 provided 10 or more full answer sets, totaling 178 (60.3%) of all analyzed answers. Furthermore, one-third (n=12) of all LTCFs provided less than 5 full answer sets.

The willingness to change behavior was higher in the serious game group (82% [119/145], 95% CI 76%-88% versus 56% [84/150], 95% CI 48%-64%; *P*<.001), with an unadjusted odds ratio of 3.60 (95% CI 2.11-6.13; *P*<.001). After adjusting for professional category and baseline knowledge, using a random effects logistic regression model with LTCF as a random effect, the magnitude of the effect increased slightly, with an odds ratio of 3.86 (95% CI 2.18-6.81; *P*<.001). The effect was not significantly affected by professional category (*P*=.46) or baseline knowledge (*P*=.52). The ICC of 0.07 (95% CI 0.01-0.33) suggests little correlation of responses within individual LTCFs, although the chi-bar-square test showed that there was good evidence against the null hypothesis of no random effects (*P*=.046). A sensitivity analysis performed by excluding answers coming from LTCFs with <10 answers yielded an unadjusted odds ratio of 2.42 (95% CI 1.25-4.68; *P*=.009) and an adjusted odds ratio of 2.54 (95% CI 1.25-5.13; *P*=.01).

The factors underlying the willingness or lack thereof to change IPC behavior are detailed in Tables 2 and 3. The factors that could have led participants to change their behavior can be found in Table 4.

**Table 2:** Factors underlying the willingness to change infection prevention and control behavior.

Factors	Control (n=84), n (%)	Serious game (n=119), n (%)
The feeling of playing an important role in the common effort against the epidemic	60 (71.4)	82 (68.9)
The information given in the training material	55 (65.5)	74 (62.2)
The probability of infecting a relative	42 (50.0)	58 (48.7)
One should follow the procedures	38 (45.2)	57 (47.9)
Other	1 (1.2)	3 (2.5)

**Table 3:** Factors underlying the lack of willingness to change infection prevention and control behavior.

Factors	Control (n=66), n (%)	Serious game (n=26), n (%)
I already apply all these guidelines	62 (93.9)	23 (88.5)
This material was not in line with my situation	6 (9.1)	4 (15.4)
The material I have just seen was not helpful	0 (0.0)	0 (0.0)
I do not believe these measures to be useful	0 (0.0)	0 (0.0)
I disagree with these measures	0 (0.0)	0 (0.0)
Other	3 (4.6)	3 (11.5)

**Table 4:** Factors that could have brought about a change in infection prevention and control behavior.

Factors	Control (n=66), n (%)	Serious game (n=26), n (%)
A better understanding of the reasons underlying the recommendations	19 (28.8)	7 (26.9)
A greater probability of infecting a relative	12 (18.2)	8 (30.8)
The feeling of having an important role in the common effort against the epidemic	28 (42.4)	12 (46.2)
Another reason	19 (28.8)	5 (19.2)
Nothing—I could not have been convinced by any argument	2 (3.0)	2 (7.7)

**Figure 10:**
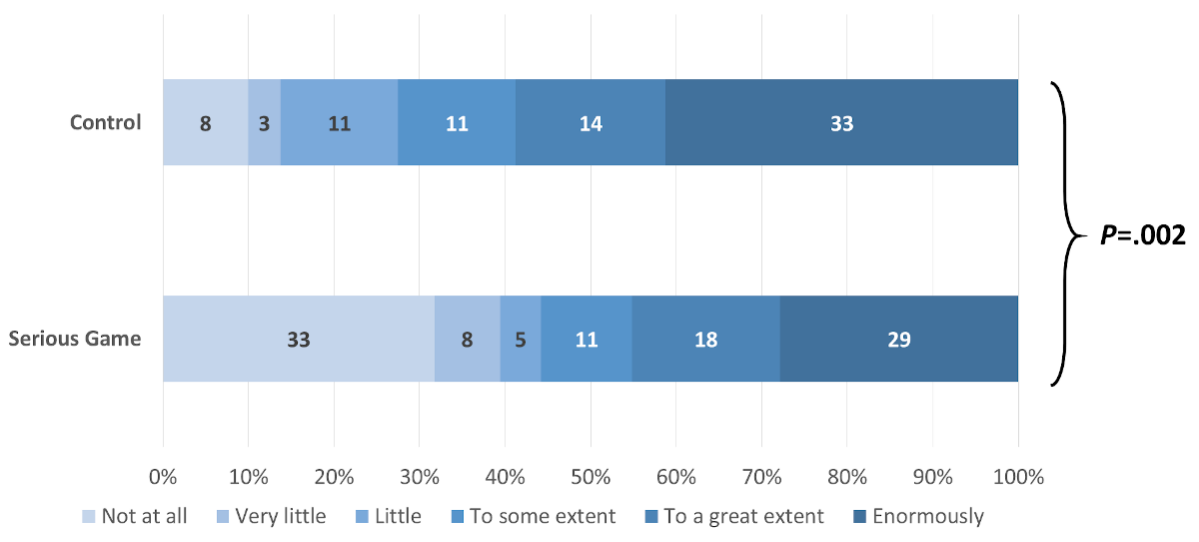
Magnitude of the willingness to change workplace disinfection behavior.

**Figure 11:**
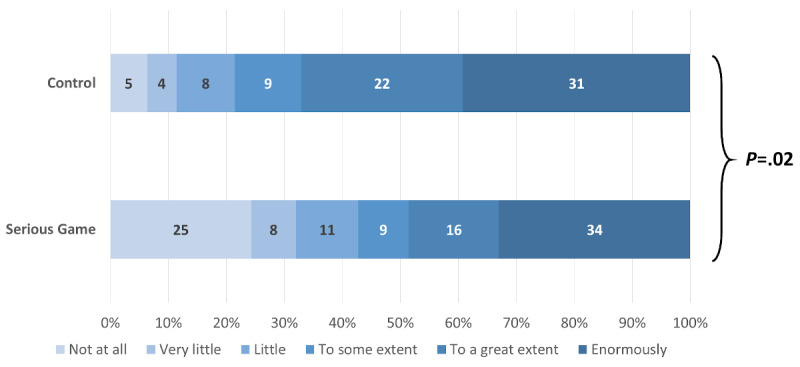
Magnitude of the willingness to change face mask handling behavior.

**Figure 12:**
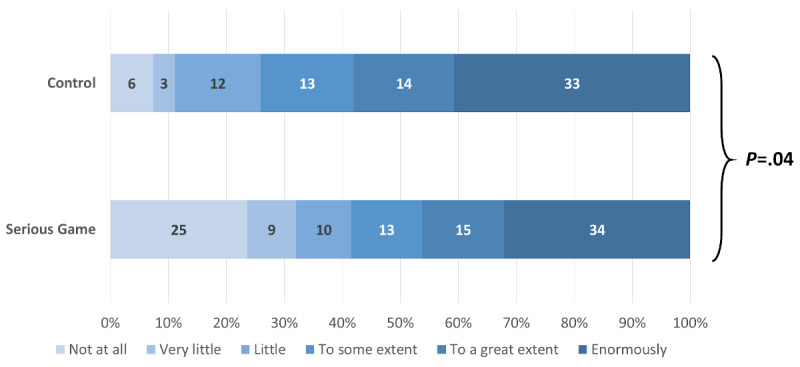
Magnitude of the willingness to protect oneself from asymptomatic people.

Among the participants who answered that they were willing to change their IPC behavior after following the learning material, those in the control group exhibited a higher intensity in their willingness to adopt the recommended behaviors (4.88, 95% CI 0.56-9.22; *P*=.03). Adjustment for baseline knowledge, professional status, and LTCF slightly increased the effect, which did not change direction and remained statistically significant (4.98, 95% CI 0.85-9.10; *P*=.02). When analyzed separately, there were no significant differences between the control and intervention group in six IPC domains: not going to work if symptomatic (*P*=.07), protection from both colleagues and patients (*P*=.09), donning sequences with and without risk of aerosolization (*P*=.54 and *P*=.72, respectively), more frequently changing gloves (*P*=.26), and practicing hand hygiene (*P*=.33; Multimedia Appendix 6). However, participants in the control group felt significantly more concerned regarding workplace disinfection (*P*=.002; Figure 10), handling of the face mask (*P*=.02; Figure 11), and protecting themselves from asymptomatic people (*P*=.04) than participants in the intervention group (Figure 12).

There was no overall difference between the groups in the intention of participants to change their use of specific PPE items after studying the IPC material (0.56, 95% CI –0.36 to 1.48; *P*=.23). Discarding the question about the use of N95 respirators did not affect this result (0.45, 95% CI –0.24 to 1.14; *P*=.20). There was no significant difference when PPE items were analyzed separately (surgical masks, *P*=.18; N95 respirators, *P*=.42; ocular protections, *P*=.22; gloves, *P*=.47; Multimedia Appendix 7).

## Discussion

### Principal Results

After following an online learning path, most participants reported that they were willing to change their IPC behavior. The serious game “Escape COVID-19” was, however, significantly more successful at inducing that change than the simple presentation of IPC guidelines. As this game was created through a theory-driven development process [24,25,50], its success at achieving the intended outcome reinforces the conclusions made by Gentry et al [29] in their recent systematic review, in which they called for further research using such methods.

Factors underlying the willingness to change IPC behavior were very similar between groups, with the feeling of playing an important role against the epidemic being most prominent, superseding even the information given in the training material. This could explain, at least in part, the success of the serious game over the standard IPC guidelines. Indeed, the “exposition” and “engagement” elements of Nicholson’s RECIPE for meaningful gamification [25] were extensively used in the development of “Escape COVID-19” [24]. The exposition element is related to the creation of a meaningful narrative in the serious game. This was achieved by having the player go through steps they would usually encounter during the course of a regular work day, including informal times such as breaks or meals. The engagement element is two tiered, the first tier being linked to social engagement and the second to the concept of “flow.” This latter concept relates to a progressive increase in the game’s difficulty to avoid disinterest [58]. The former, social engagement, is usually achieved by the creation of multiplayer modes. Although “Escape COVID-19” lacks such a mode, it nevertheless takes this element into account by having the player make decisions that would, in real life, affect other people. To further strengthen the importance of these elements, analyzing the factors that could have motivated a change in IPC behavior revealed that the feeling of having an important role in the common effort against the epidemic was the leading factor that could have convinced participants to adapt their practices. This finding is in line with the driving force of the personal locus of control and the receptiveness to learning about and engaging in new behavior in the health care field [59].

The factors underlying the lack of willingness to change IPC behavior were also similar between groups. The vast majority of participants unwilling to change answered that they were already applying all the guidelines they had been presented with. As the serious game contained little material other than that presented in the standard IPC guidelines, it might therefore be surmised that the serious game was also more successful in conveying key IPC messages. Nevertheless, the magnitude of the intention to change IPC practices was significantly higher for some specific aspects in the fewer participants who were willing to change their IPC practices after following the control materials. Our hypothesis is that these participants might have been looking for information regarding some specific IPC aspects and were from the start highly motivated to change their practices according to relevant and up-to-date IPC guidelines, regardless of the way the information was presented. Another explanation is that, even though the serious game was more engaging, it was not designed to give in-depth explanations regarding specific IPC measures. This might have contributed to this difference, even though the proportion of participants answering that “a better understanding of the reasons underlying the recommendations” would have made them more willing to change their IPC practices was similar between groups.

No participant answered that they disagreed with the IPC guidelines or that they did not believe such measures to be useful. Given the current inclination for fake news [60-62] and conspiracy theories [63-65], this is rather reassuring, even more so as some participants were not HCWs but members of administrative, catering, and hospitality staff. More reassuring still is that the analysis of the factors that could have brought about a change in IPC behavior showed that only very few participants answered that they could not have been convinced by any argument.

### Limitations and Strengths

This study has several limitations. First, even though the probability of executing an action is strongly linked to the intention of performing it, one can hardly be certain that LTCF employees claiming they are willing to change their IPC behavior will actually change it. Field observations would be necessary to ascertain this aspect, along with a different study design as both groups ultimately accessed the serious game in this study. Another important limitation is that we did not reach the sample size we had expected [34]. Indeed, while we had hoped for at least 800 participants, the actual number of accounts activated by LTCF employees was rather lower. This low figure raises many questions and hypotheses. Indeed, while at least a complete answer set was obtained for each individual LTCF, less than 10 complete answer sets were given by more than three-quarters (28/36) of all LTCFs under the jurisdiction of the public health authorities of Geneva, with almost half of those (12/28) giving less than 5 complete answer sets. It might therefore be assumed that, while all LTCF managers received the information regarding the study, many decided not to forward it to their employees. This was an unexpected finding; however, this study was not designed to assess the reasons underlying this decision. Hypotheses can however be drawn, some of which are more concerning than others. Among the least concerning, fear of overloading already overworked LTCF employees with information, an insufficient number of reminders, or the simple lack of regularly updated mailing lists could partly explain the low participation rate witnessed in many LTCFs. Moreover, some managers might have felt that the material, which originated from a university hospital, was not in line with their situation. However, this hypothesis is challenged by the fact that this impression, though asked for, was reported by 10 participants only. Lack of eHealth literacy is probably not to blame for the lack of participation. Indeed, LTCF employees increasingly use digital devices in the course of their work [66], and recent surveys have shown that eHealth literacy was rather high in HCWs [67]. Among the most disturbing hypotheses, a low level of concern of some LTCF managers, a potential mistrust of health authorities or the institution authoring the game, or even of IPC guidelines, cannot be ruled out. The will to avoid spreading information that could lead to an increase in the use of PPE items and, therefore, to an increase in material costs, seems unlikely. Regardless of the reason, the ability of health authorities to successfully convey critical messages by efficient vectors to LTCF employees should be assessed specifically to solve potential communication issues. The creation of IPC focal points in each LTCF is a path that could be explored.

Despite these limitations, this study also has some strengths, among which the fully automated randomization process, the triple-blinding, and the originality of the material could be mentioned. Finally, despite the lower-than-expected participation rate, the presence of a control group has enabled us to limit certain biases, as one could also hypothesize that only the most motivated LTCF employees would have participated, thereby creating a selection bias and limiting the interpretation of our findings.

### Perspectives

This serious game might not be equally effective in all populations, and IPC messages might differ from one region to another. By virtue of its flexible design, “Escape COVID-19” can be updated rather easily and should now be tested on other populations. It has been fully translated into English and is in the process of being translated into German and Italian to allow its deployment at the Swiss national level. To enhance its visibility, publicizing actions similar to those used to promote another recently developed serious game (“COVID-19 – Did You Know?) should be considered [68]. Currently, “Escape COVID-19” is freely available to play online [53], and the corresponding author can be contacted at any time to obtain a SCORM package in either French or English, while translation into other languages is pending.

### Conclusion

Among LTCF employees, the serious game “Escape COVID-19” was more successful than standard IPC material in inspiring the willingness to adopt COVID-19–safe IPC behavior.

## Appendix

Multimedia Appendix 1 Email template.

Multimedia Appendix 2 Email received upon account creation.

Multimedia Appendix 3 First questionnaire.

Multimedia Appendix 4 Second questionnaire.

Multimedia Appendix 5 Original versions of the screen captures, in French.

Multimedia Appendix 6 Graphical results of questions regarding infection prevention and control domains (based on the 6-point Likert scale).

Multimedia Appendix 7 Graphical results of questions regarding specific personal protective equipment items (based on the 5-point Likert scale).

Multimedia Appendix 8 CONSORT-EHEALTH checklist (V 1.6.1).

## Abbreviations

CHERRIES: Checklist for Reporting Results of Internet E-Surveys
CONSORT: Consolidated Standards of Reporting Trials
HCW: health care worker
ICC: intraclass correlation coefficient
IPC: infection prevention and control
LTCF: long-term care facility
PPE: personal protective equipment
SCORM: Shareable Content Object Reference Model
