# Supplementary material for: Impact of a Serious Game (Escape COVID-19) on the Intention to Change COVID-19 Control Practices Among Employees of Long-term Care Facilities: Web-Based Randomized Controlled Trial
Source: J Med Internet Res. 2021 Mar 25;23(3):e27443. doi: 10.2196/27443 (PMC7996198; doi:10.2196/27443)
Supplement: Multimedia Appendix 3 [file jmir_v23i3e27443_app3.pdf]

**Multimedia Appendix 3.** First questionnaire, designed to gather demographic data and assess the baseline knowledge level of nursing home employees.

| Page | Field | Original Question | English Translation |
|---|---|---|---|
| 1 | Demographics | Vous êtes:<br><br>- Un homme<br>- Une femme | You are:<br><br>- A man<br>- A woman |
| | | Quel est votre âge? | What is your age? |
| | | Vous faites principalement partie du personnel:<br><br>- Médical<br>- Soignant<br>- Administratif/support<br>- Autre [a] | You are mainly part of the:<br><br>- Medical staff<br>- Nursing staff<br>- Administrative/support staff<br>- Other [a] |
| | | Vous êtes:[b]<br><br>- Infirmier.e<br>- Assistant.e en soins et santé communautaire<br>- Aide-soignant.e<br>- Physiothérapeute<br>- Autre [a] | You are:[b]<br><br>- Nurse<br>- Nursing assistant<br>- Health care assistant<br>- Physiotherapist<br>- Other [a] |
| | | Vous êtes en contact avec des patients:[c]<br><br>- Très fréquemment<br>- Assez fréquemment<br>- Peu fréquemment<br>- Quasiment jamais | You are in contact with patients :[c]<br><br>- Very frequently<br>- Quite frequently<br>- Seldom<br>- Almost never |
| | | Depuis combien d'années travaillez-vous dans le domaine de la santé? | For how many years have you worked in health care? |
| 2[d] | Baseline knowledge | Vous vous apprêtez à entrer dans la chambre d'un patient COVID-19 **qui n'est pas sous CPAP** pour lui prodiguer des soins, quels | You are about to enter a room to care for a COVID-19 patient **who is not receiving CPAP treatment**. What protective equipment should you wear? |

| | | | |
|---|---|---|---|
| | | équipements de protection sont nécessaires?<br><br>- Masque médical<br>- Surblouse<br>- Gants<br>- Tablier de soins<br>- Protection oculaire<br>- Masque ultrafiltrant (FFP2) | - Face mask<br>- Gown<br>- Gloves<br>- Protective apron<br>- Eye protection<br>- N95 mask |
| | | Vous vous apprêtez à entrer dans la chambre d'un patient COVID-19 **qui est sous CPAP** pour lui prodiguer des soins, quels équipements de protection sont nécessaires?<br><br>- Masque médical<br>- Surblouse<br>- Gants<br>- Tablier de soins<br>- Protection oculaire<br>- Masque ultrafiltrant (FFP2) | You are about to enter a room to care for a COVID-19 patient **who is under CPAP**. What protective equipment should you wear?<br><br>- Face mask<br>- Gown<br>- Gloves<br>- Protective apron<br>- Eye protection<br>- N95 mask |
| | | Quelle est durée médiane d'incubation du COVID-19 (en jours)?<br><br>- 2-3 jours<br>- 4-6 jours<br>- 7-10 jours<br>- 11-14 jours | What is the median incubation time of COVID-19 (in days)?<br><br>- 2-3 days<br>- 4-6 days<br>- 7-10 days<br>- 11-14 days |
| | | Parmi les situations suivantes, lesquelles doivent conduire à porter un masque ultrafiltrant (FFP2) chez un patient COVID-19 (confirmé ou suspecté)?<br><br>- Auscultation d'un patient COVID-19 qui tousse<br>- Traitements par nébulisation<br>- Oxygénation à haut débit<br>- Séance de physiothérapie de rééducation à la marche<br>- Oxygénothérapie nasale à moins de 2L/min | In which of the following situations should you wear an N95 respirator mask when taking care of patient with a COVID-19 infection (confirmed or suspected)?<br><br>- Auscultation of a coughing COVID-19 patient<br>- Nebulization therapy<br>- High flow oxygen therapy<br>- Gait rehabilitation physiotherapy session<br>- Nasal oxygen therapy (flow less than 2L/min) |

| | | | |
|---|---|---|---|
| | | Parmi les suivantes, dans quelles situations le port de protections oculaires est-il recommandé ?<br><br>- En cas de contact anticipé avec un liquide biologique par éclaboussure<br>- En cas de contact de proximité avec un patient qui présente de symptômes respiratoires, même en l'absence de diagnostic de COVID-19<br>- En tout temps dans la chambre d'un patient COVID-19<br>- En tout temps dans toute situation dans tous les lieux de l'établissement de soins | In which of the following situations should you wear eye protection?<br><br>- If contact with a biological liquid (splashing) is anticipated<br>- In case of close contact with a patient with respiratory symptoms, even without a diagnosis of COVID-19<br>- Anytime when in the room of a COVID-19 patient<br>- At all times in any situation in all areas of the care facility |
| 2e | Baseline knowledge | Dans les lieux communs de l'institution, quels équipements de protection doivent être portés lorsque la distance de 1.5 m ne peut pas être respectée?<br><br>- Le masque médical<br>- Les gants<br>- Le masque ultrafiltrant (FFP2)<br>- Les protections oculaires<br>- La surblouse | In the common areas of the institution, what protective equipment should be worn when the distance of 1.5 m cannot be respected?<br><br>- A face mask<br>- Gloves<br>- An N95 respirator<br>- Eye protection<br>- A protective gown |
| | | Parmi les suivantes, dans quelles situations le port de protections oculaires est-il recommandé ?<br><br>- En cas de contact anticipé avec un liquide biologique par éclaboussure<br>- En cas de contact de proximité avec un patient qui présente de symptômes respiratoires, même en l'absence de diagnostic de COVID-19<br>- En tout temps dans la chambre d'un patient COVID-19 | In which of the following situations should you wear eye protection?<br><br>- If contact with a biological liquid (splashing) is anticipated<br>- In case of close contact with a patient with respiratory symptoms, even without a diagnosis of COVID-19<br>- Anytime when in the room of a COVID-19 patient<br><br>At all times in any situation in all areas of the care facility |

| | | | |
|---|---|---|---|
| | | En tout temps dans toute situation dans tous les lieux de l'établissement de soins | |
| | | Si vous vous trouvez dans la même pièce qu'un patient atteint de COVID-19 qui est sous CPAP, vous devez porter un masque ultrafiltrant (FFP2)<br><br>- Vrai<br>- Faux | If you are in a room with a COVID-19 patient who is under CPAP, you must wear an N95 respirator mask<br><br>- True<br>- False |
| | | Le bio-nettoyage standard de la chambre d'un patient COVID-19 nécessite l'utilisation d'un désinfectant standard (Des-sur®)<br><br>- Vrai<br>- Faux | Standard bio-cleaning of a COVID-19 patient's room requires the use of a standard disinfectant (Des-sur®)<br><br>- True<br>- False |
| 3 | Symptoms and screening | Si j'ai des symptômes compatibles avec le COVID-19 :<br><br>- J'attends de voir l'évolution et en cas de persistance des symptômes je vais me faire tester<br>- Je vais me faire tester le jour même<br>- Si les symptômes sont légers, j'attends que les symptômes passent et je continue de travailler sans faire de test | If I have symptoms compatible with COVID-19:<br><br>- I monitor their evolution and get tested if the symptoms persist<br>- I get tested right away (on the same day)<br>- If the symptoms are mild, I wait for the symptoms to recede and continue to work without getting tested |
| | | Parmi les symptômes suivants, quels sont ceux qui devraient vous conduire à faire un test de dépistage du COVID-19?<br><br>- Rhume<br>- Eruption cutanée<br>- Toux<br>- Maux de gorge<br>- Perte de l'odorat<br>- Maux de tête | Which of the following symptoms should prompt you to get tested for COVID-19?<br><br>- Symptoms compatible with a "cold"<br>- Skin rash<br>- Cough<br>- Sore throat<br>- Loss of smell<br>- Headache |

[a]Answering “other” allows the participant to enter free text in a specific field.

[b]Displayed only to participants who identify as part of the “nursing staff.”

[c]Displayed only to participants who identify as part of the “administrative/support staff” or as “other.”

[d]These questions are only displayed to members of the medical or nursing staff.

[e]These questions are only displayed to participants who identify as part of the “administrative/support staff” or as “other.”
