# Supplementary material for: Impact of a Serious Game (Escape COVID-19) on the Intention to Change COVID-19 Control Practices Among Employees of Long-term Care Facilities: Web-Based Randomized Controlled Trial
Source: J Med Internet Res. 2021 Mar 25;23(3):e27443. doi: 10.2196/27443 (PMC7996198; doi:10.2196/27443)
Supplement: Multimedia Appendix 4 [file jmir_v23i3e27443_app4.pdf]

Multimedia Appendix 4. Second questionnaire, designed to determine the intention of changing prevention infection and control practices in nursing home employees.

Original Question	English Translation
Après avoir vu ce matériel de formation/information, allez-vous modifier certaines de vos pratiques de prévention de l'infection?<br><br>- Oui<br>- Non	After seeing this training / information material, are you going to change any of your infection prevention practices?<br><br>- Yes<br>- No
Quels domaines ces changements vont-ils concerner?[a,b]<br><br>- Le fait de ne pas aller au travail si vous présentez des symptômes compatibles avec le COVID-19<br>- Le fait de vous protéger autant de vos collègues que de vos patients<br>- La séquence d'habillage lors de procédures AVEC risque d'aérosolisation<br>- La séquence d'habillage lors de procédures SANS risque d'aérosolisation<br>- Le fait de changer plus fréquemment de gants non stériles<br>- Le fait de vous désinfecter les mains plus fréquemment<br>- Le fait de désinfecter votre place de travail<br>- Le fait de manipuler le masque médical avec plus de précautions<br>- Le fait de vous protéger également des personnes asymptomatiques	What areas will these changes affect? [a,b]<br><br>- Not going to work if you have symptoms compatible with COVID-19<br>- Protecting yourself from both your colleagues and your patients<br>- The donning sequence when dealing with procedures CARRYING a risk of aerosolization<br>- The donning sequence when dealing with procedures NOT CARRYING a risk of aerosolization<br>- Changing non-sterile gloves more frequently<br>- Practicing hand hygiene more frequently<br>- Disinfecting your workplace<br>- Handling the face mask more carefully<br>- Protecting yourself from asymptomatic people as well as from symptomatic ones
Vous allez désormais employer: [a,c]<br><br>- Les masques médicaux<br>- Les masques FFP-2<br>- Les protections oculaires<br>- Les gants non stériles	You are now going to use: [a,c]<br><br>- Face masks<br>- N95 respirator masks<br>- Eye protections<br>- Non-sterile gloves
Qu'est ce qui a grandement participé à votre intention de modifier vos pratiques? [a]	Which of these elements greatly contributed to your intention to modify your practices? [a]<br><br>- The information given in the training material

- L'information contenue dans le matériel de formation<br>- Le sentiment de jouer un rôle important dans l'effort commun contre l'épidémie<br>- La probabilité de contaminer un proche<br>- Il faut suivre les procédures<br>- Autre [d]	- The feeling of playing an important role in the common effort against the epidemic<br>- The probability of infecting a relative<br>- One should follow the procedures<br>- Other [d]
Pour quelles raisons vos pratiques ne changeront-elles pas? [e]<br><br>- Le matériel que je viens de consulter était inadapté à ma situation<br>- J'applique déjà toutes les mesures proposées<br>- Le matériel que je viens de consulter n'était pas utile<br>- Je ne crois pas que ces mesures soient utiles<br>- Je suis en désaccord avec les mesures proposées [d]<br>- Autre [d]	Why will your practices not change? [e]<br><br>- This material was not in line with my situation<br>- I already apply all these guidelines<br>- The material I have just seen was not helpful<br>- I do not believe these measures to be useful<br>- I disagree with these measures [d]<br>- Other [d]
Qu'est-ce qui aurait pu favoriser la modification de vos pratiques?<br><br>- Mieux comprendre les raisons justifiant les recommandations<br>- Une probabilité plus importante de contaminer un proche<br>- Le sentiment que vous avez un role important dans l'effort commun contre l'épidémie<br>- Autre [d]<br>- Rien - aucun argument ne pouvait me convaincre	What could have motivated you to change your practices?<br><br>- Better understand the reasons behind the recommendations<br>- A greater probability of infecting a relative<br>- The feeling of having an important role in the common effort against the epidemic<br>- Other [d]<br>- Nothing – I could not have been convinced by any argument

[a]Question displayed only to participants who answered they were going to change their practices.

[b]Answers based on a 6-point Likert scale (from 1, "not at all," to 6, "very much").

[c]Answers based on a 5-point Likert scale (from 1, "much less," to 5, "much more").

[d]Ticking this option allows the participant to enter free text in a dedicated field.

[e]Question displayed only to participants who answered they were not going to change their practices.
