# Supplementary material for: Impact of a Serious Game (Escape COVID-19) on the Intention to Change COVID-19 Control Practices Among Employees of Long-term Care Facilities: Web-Based Randomized Controlled Trial
Source: J Med Internet Res. 2021 Mar 25;23(3):e27443. doi: 10.2196/27443 (PMC7996198; doi:10.2196/27443)
Supplement: Multimedia Appendix 5 [file jmir_v23i3e27443_app5.pdf]

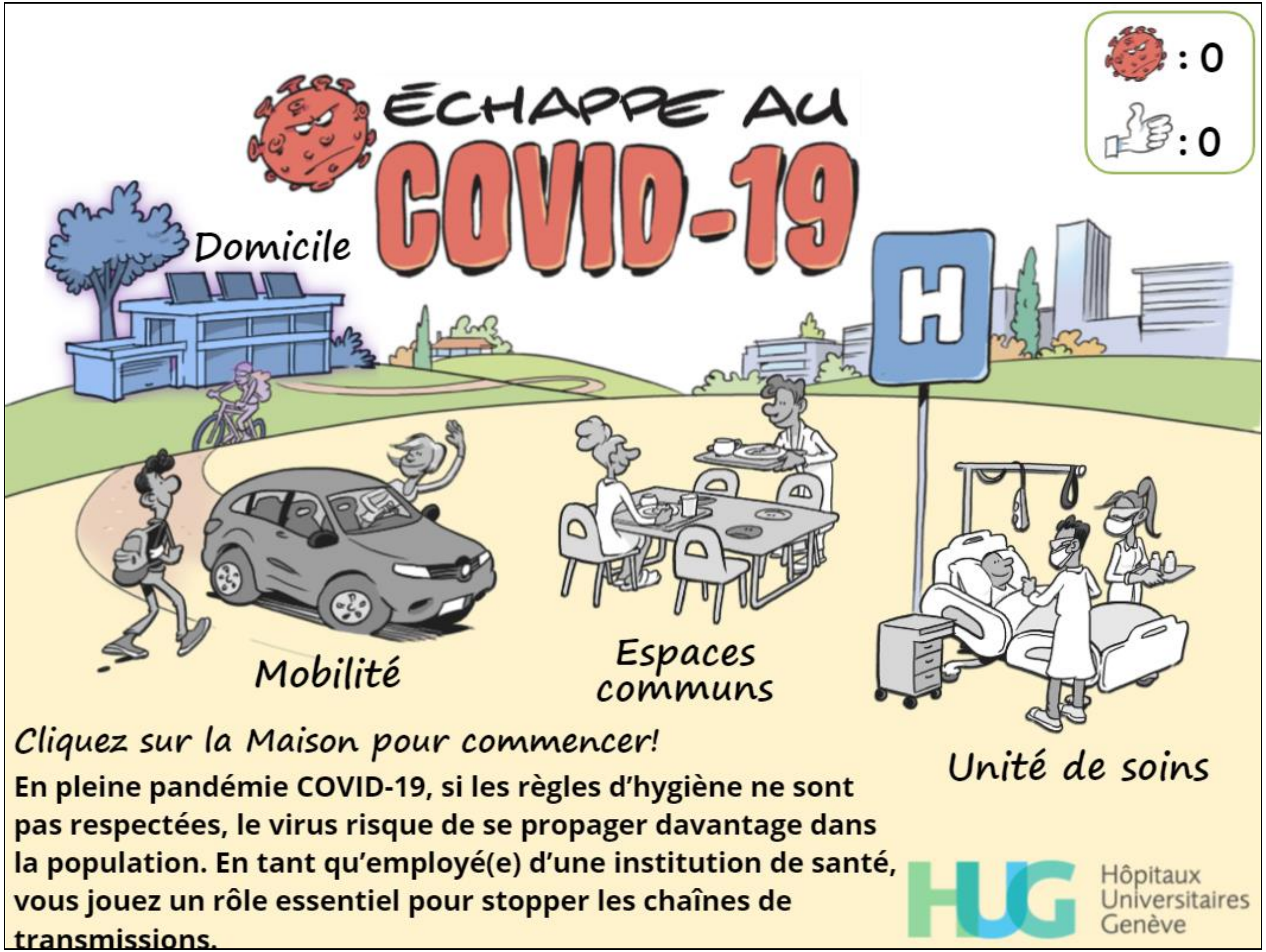

Multimedia Appendix – Figure 6 FR. Original version of the welcome screen, in French.

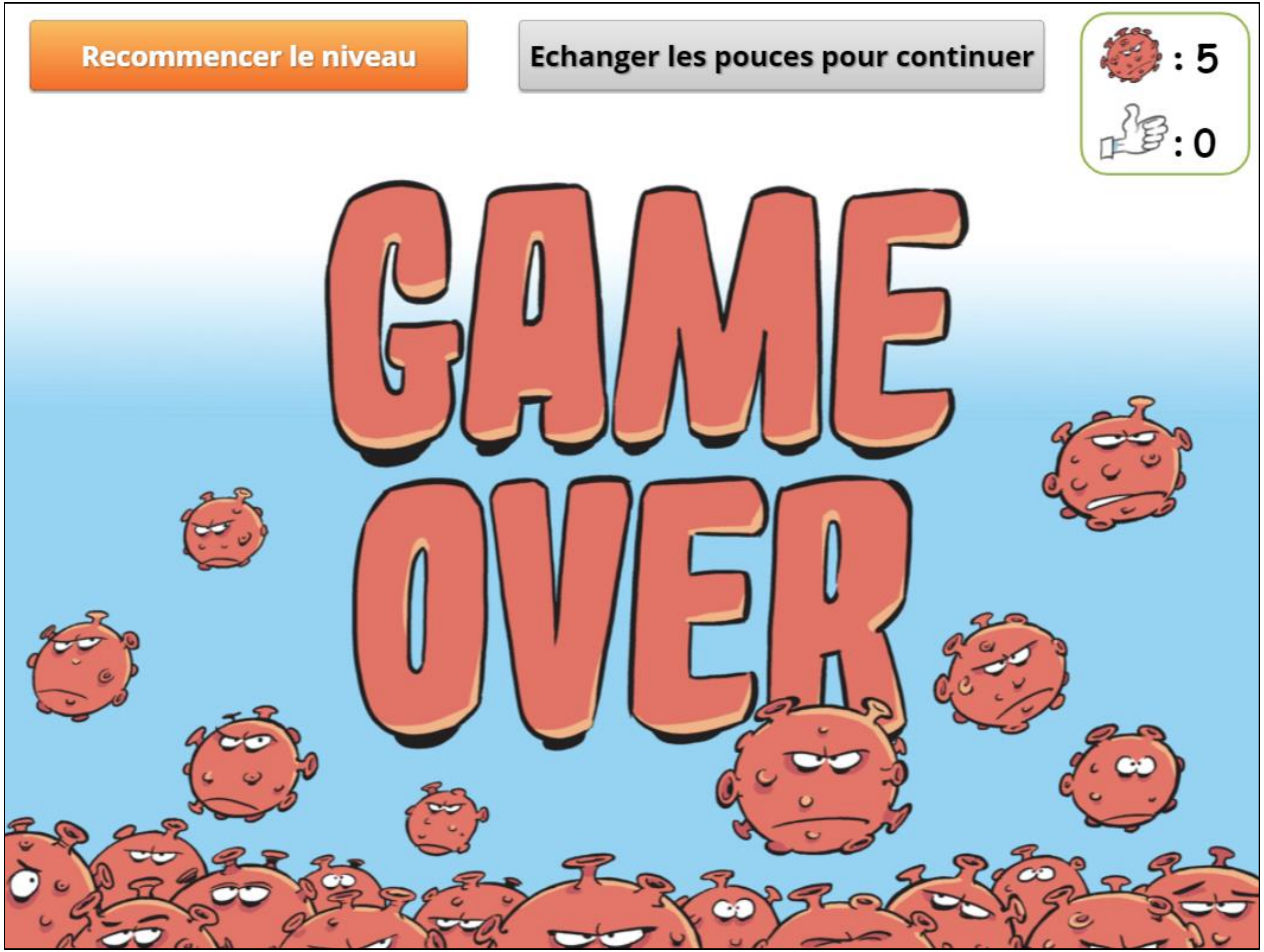

Multimedia Appendix – Figure 7 FR. Original version of the game over screen, in French.

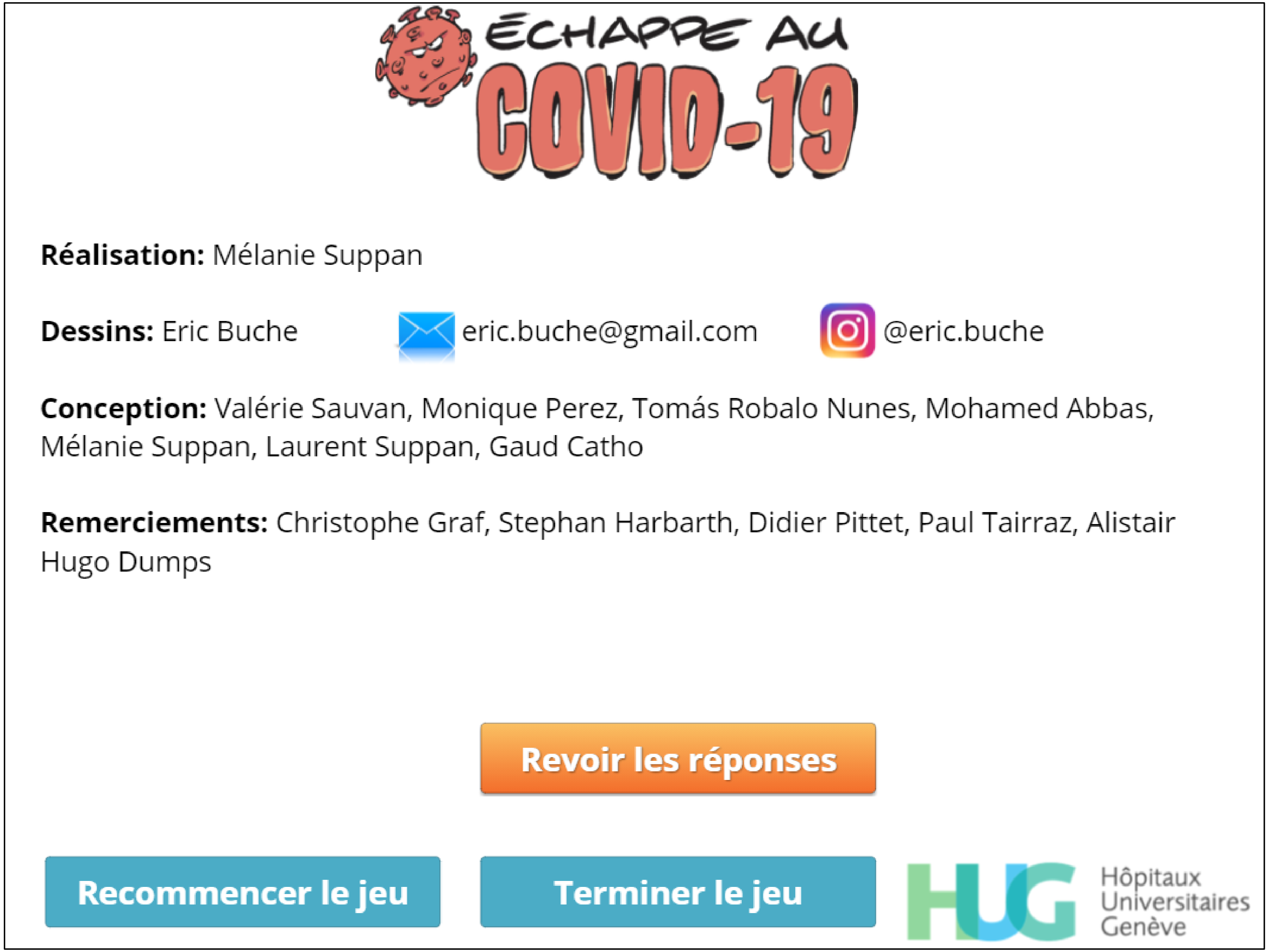

Multimedia Appendix – Figure 8 FR. Original (French) version of the screen used to let participant access the second questionnaire
