# Supplementary material for: Impact of a Serious Game (Escape COVID-19) on the Intention to Change COVID-19 Control Practices Among Employees of Long-term Care Facilities: Web-Based Randomized Controlled Trial
Source: J Med Internet Res. 2021 Mar 25;23(3):e27443. doi: 10.2196/27443 (PMC7996198; doi:10.2196/27443)
Supplement: Multimedia Appendix 6 [file jmir_v23i3e27443_app6.pdf]

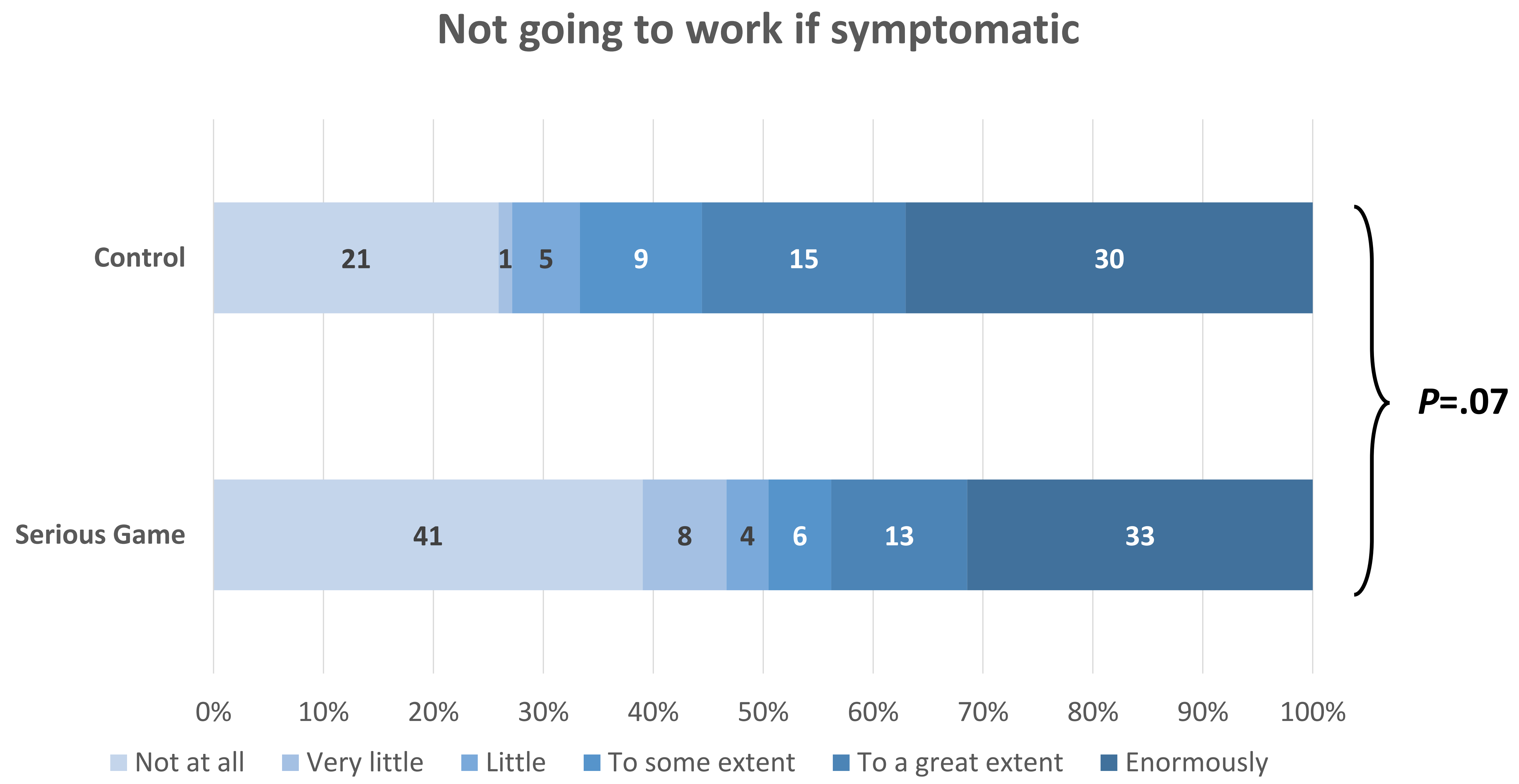
Not going to work if symptomatic
Control
21
1
5
9
15
30
Serious Game
41
8
4
6
13
33
P=.07
0%
10%
20%
30%
40%
50%
60%
70%
80%
90%
100%
Not at all
Very little
Little
To some extent
To a great extent
Enormously

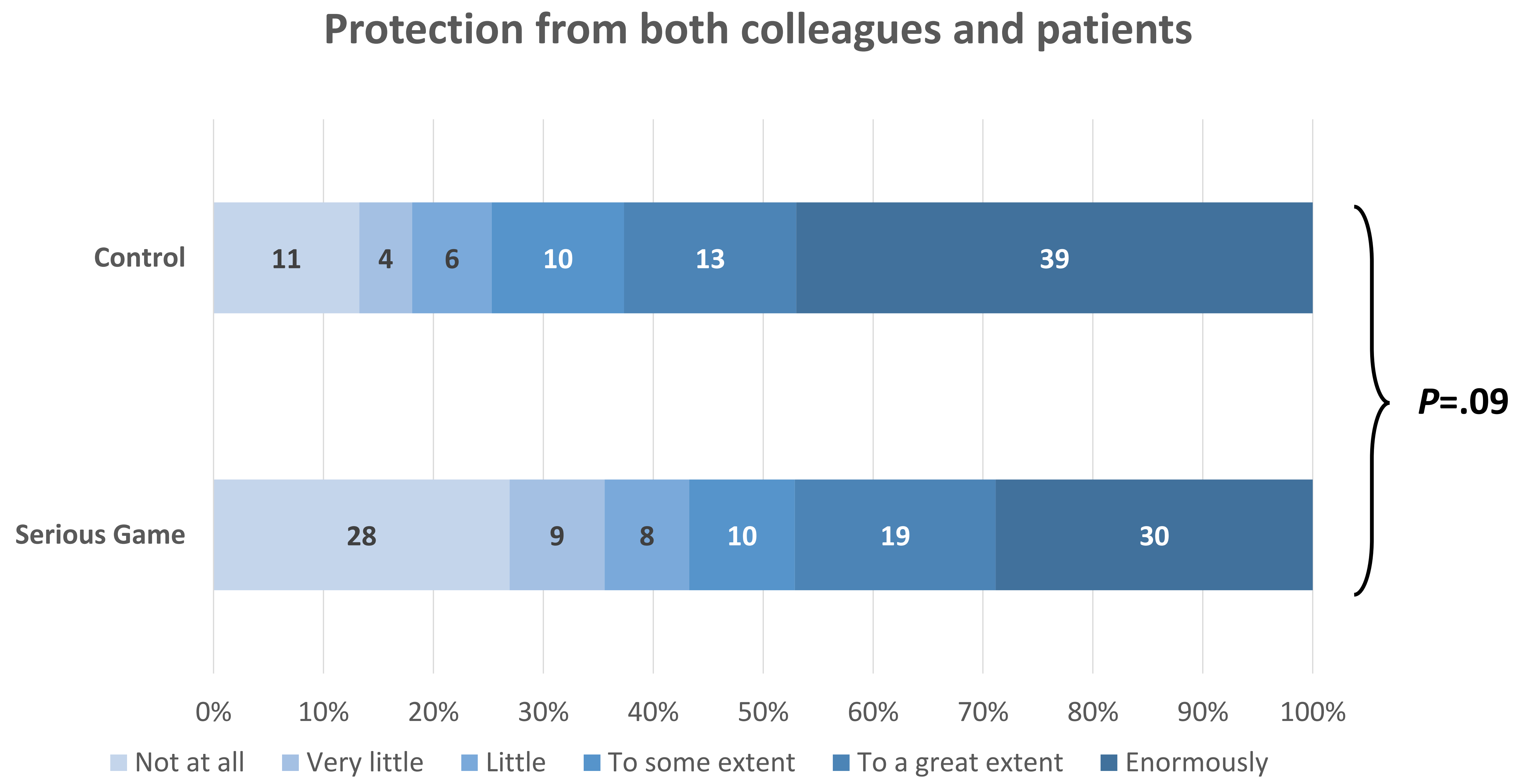

Protection from both colleagues and patients
Control
11
4
6
10
13
39
Serious Game
28
9
8
10
19
30
P=.09
0%
10%
20%
30%
40%
50%
60%
70%
80%
90%
100%
Not at all
Very little
Little
To some extent
To a great extent
Enormously

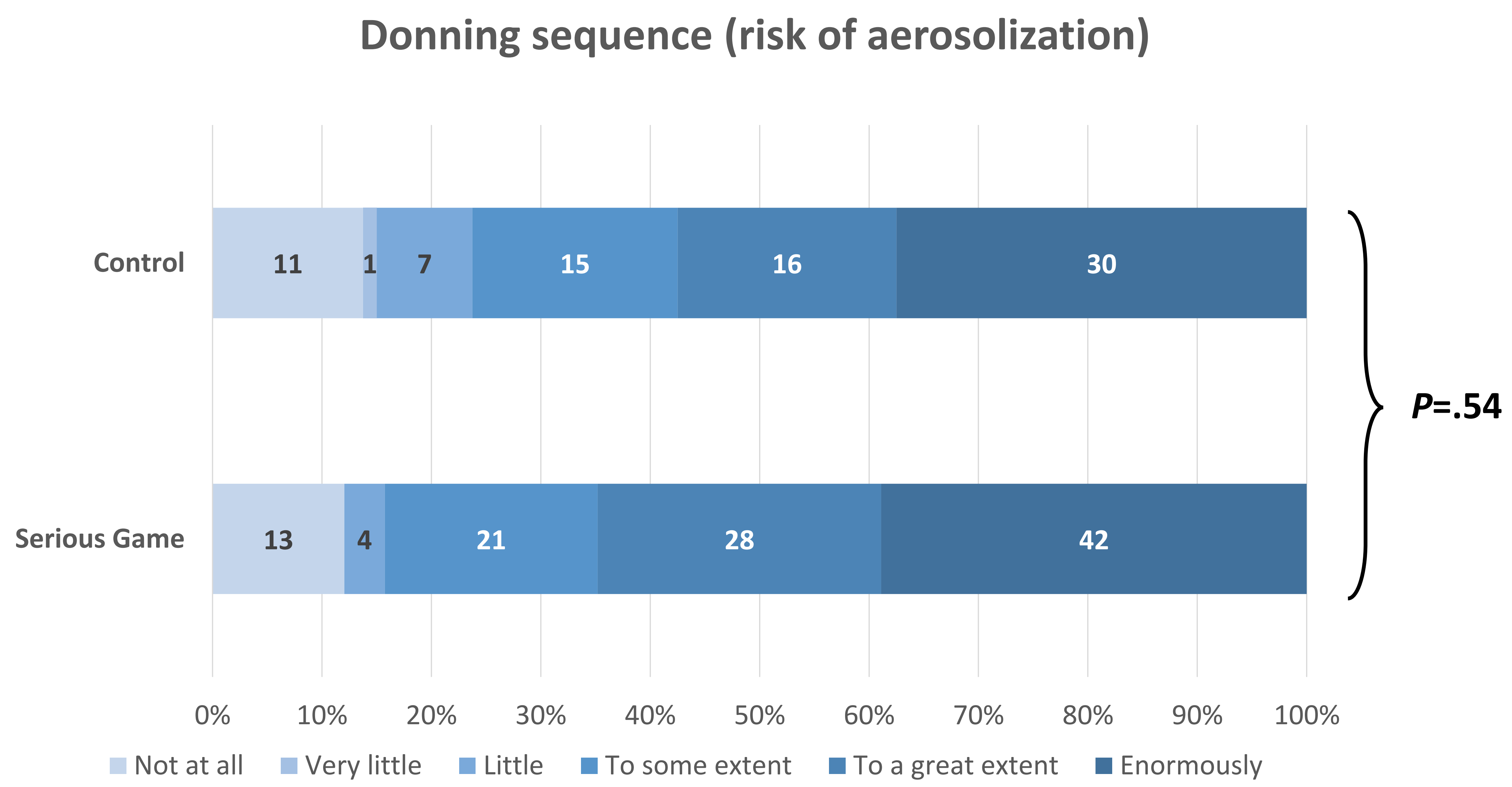

Donning sequence (risk of aerosolization)
Control
11
1
7
15
16
30
Serious Game
13
4
21
28
42
P=.54
0%
10%
20%
30%
40%
50%
60%
70%
80%
90%
100%
Not at all
Very little
Little
To some extent
To a great extent
Enormously

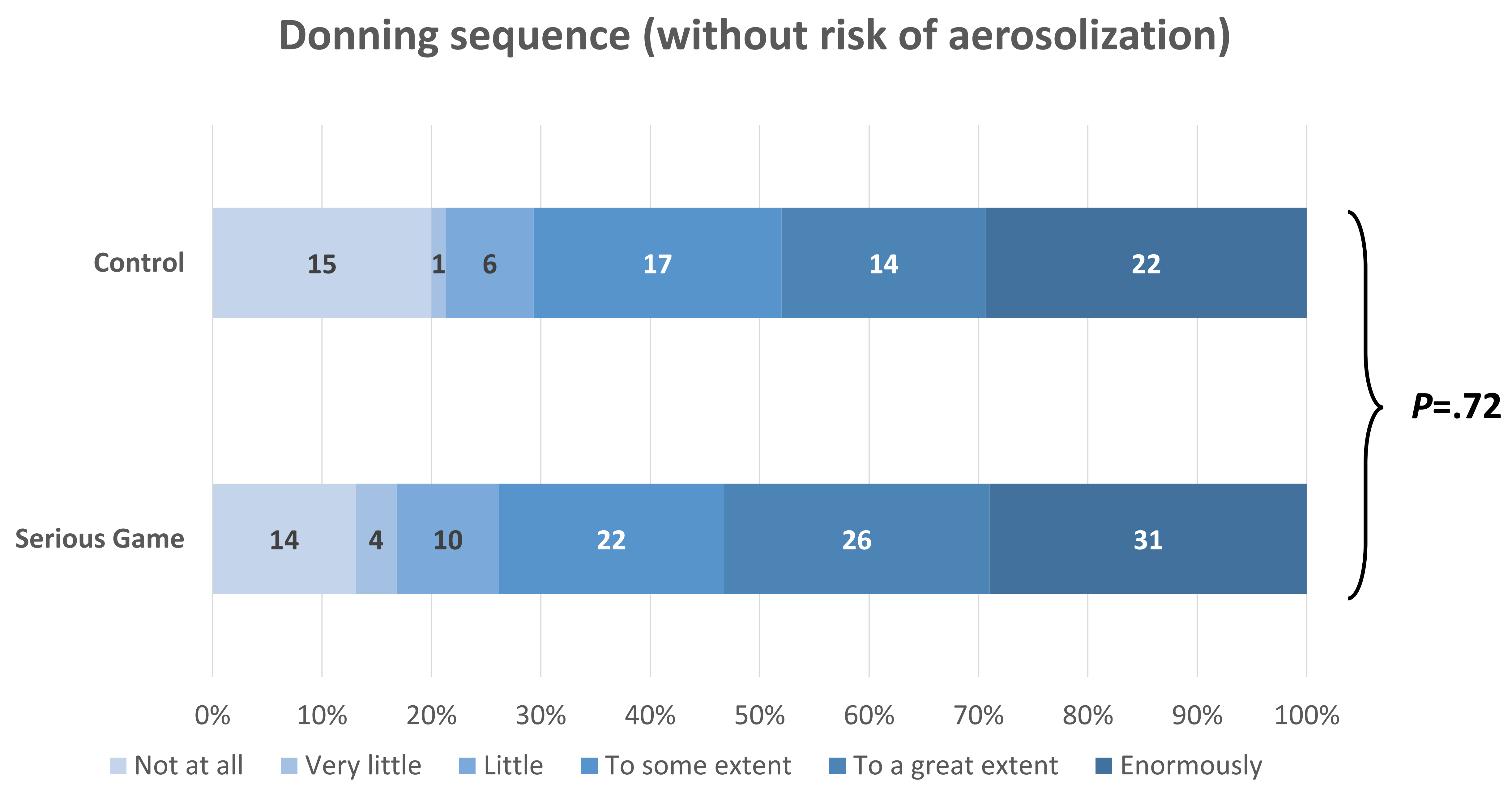
Donning sequence (without risk of aerosolization)
Control
Serious Game
15
1
6
17
14
22
14
4
10
22
26
31
P=.72
0%
10%
20%
30%
40%
50%
60%
70%
80%
90%
100%
Not at all
Very little
Little
To some extent
To a great extent
Enormously

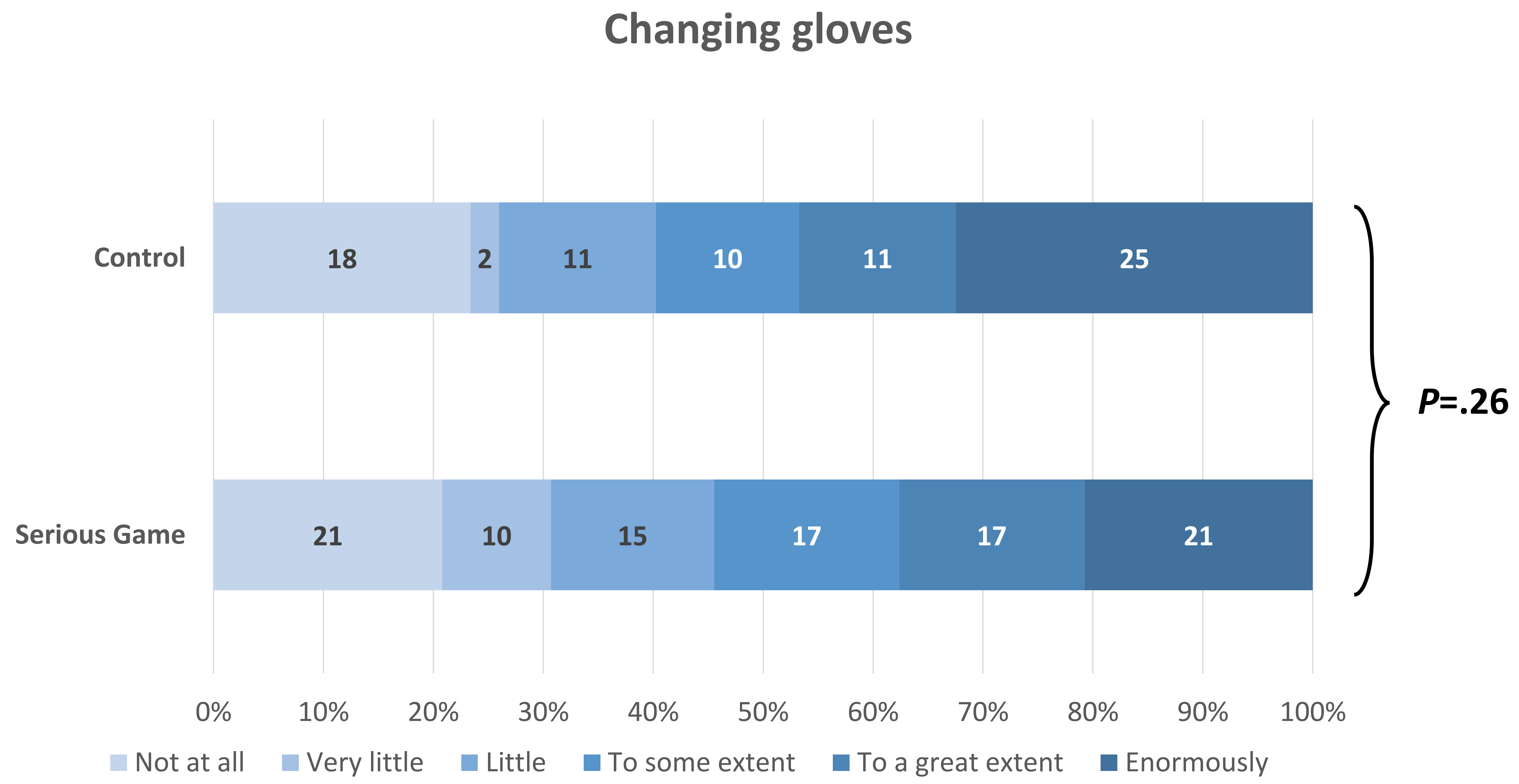
Changing gloves
Control
18
2
11
10
11
25
Serious Game
21
10
15
17
17
21
P=.26
0%
10%
20%
30%
40%
50%
60%
70%
80%
90%
100%
Not at all
Very little
Little
To some extent
To a great extent
Enormously

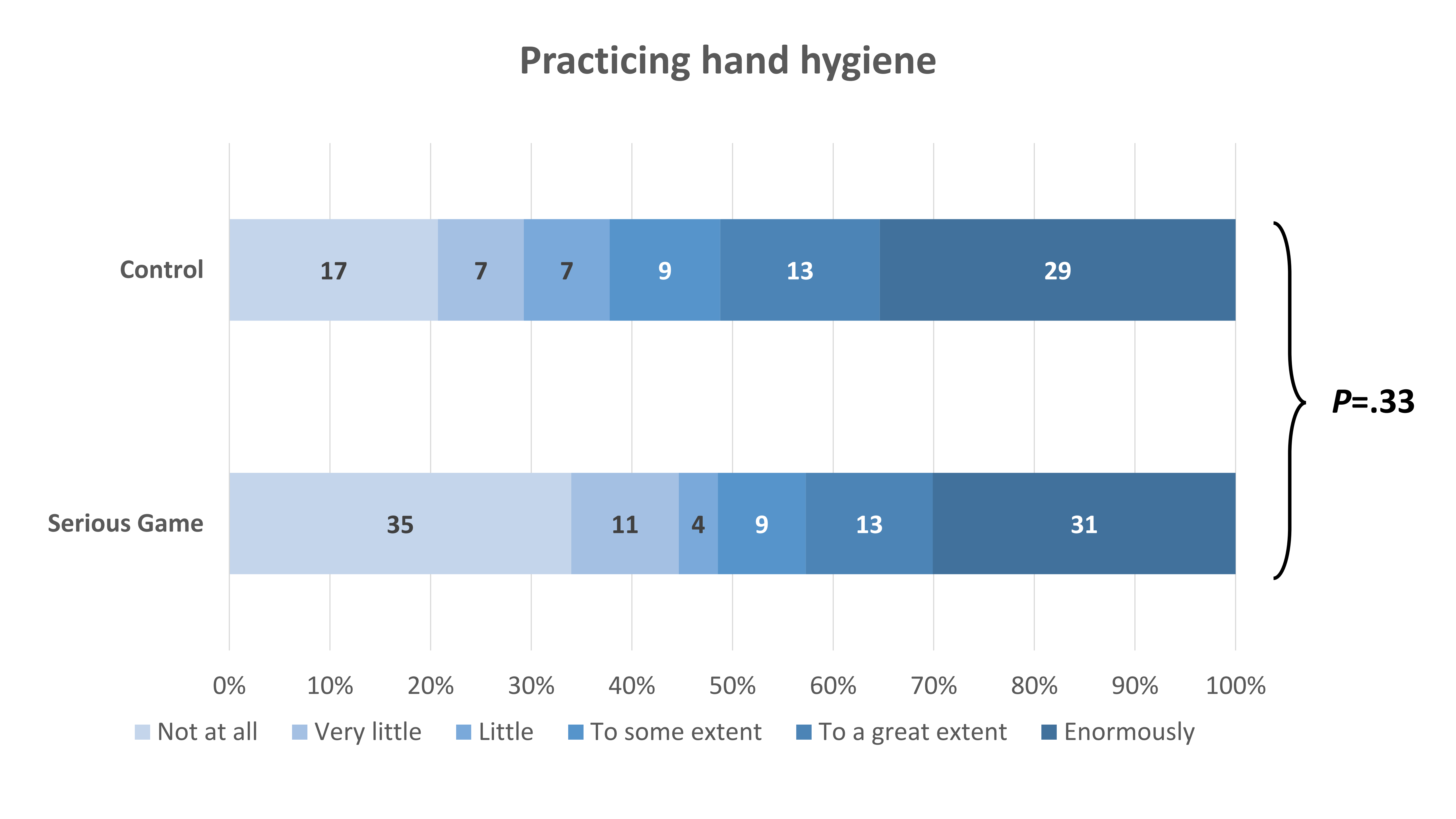

Practicing hand hygiene
Control
Serious Game
17
7
7
9
13
29
35
11
4
9
13
31
P=.33
0%
10%
20%
30%
40%
50%
60%
70%
80%
90%
100%
Not at all
Very little
Little
To some extent
To a great extent
Enormously
