# Supplementary material for: Impact of a Serious Game (Escape COVID-19) on the Intention to Change COVID-19 Control Practices Among Employees of Long-term Care Facilities: Web-Based Randomized Controlled Trial
Source: J Med Internet Res. 2021 Mar 25;23(3):e27443. doi: 10.2196/27443 (PMC7996198; doi:10.2196/27443)
Supplement: Multimedia Appendix 7 [file jmir_v23i3e27443_app7.pdf]

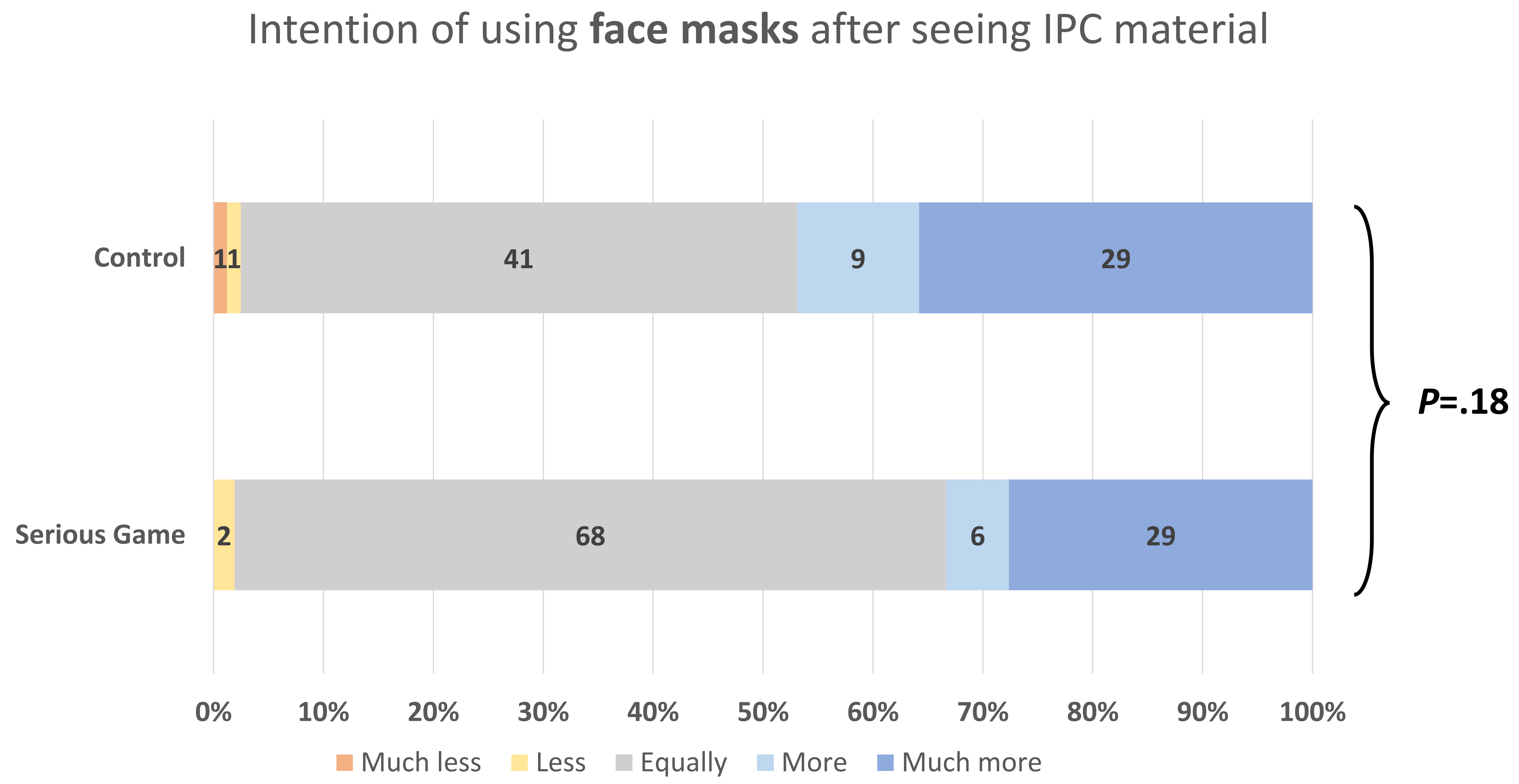

IPC: Infection Prevention and Control

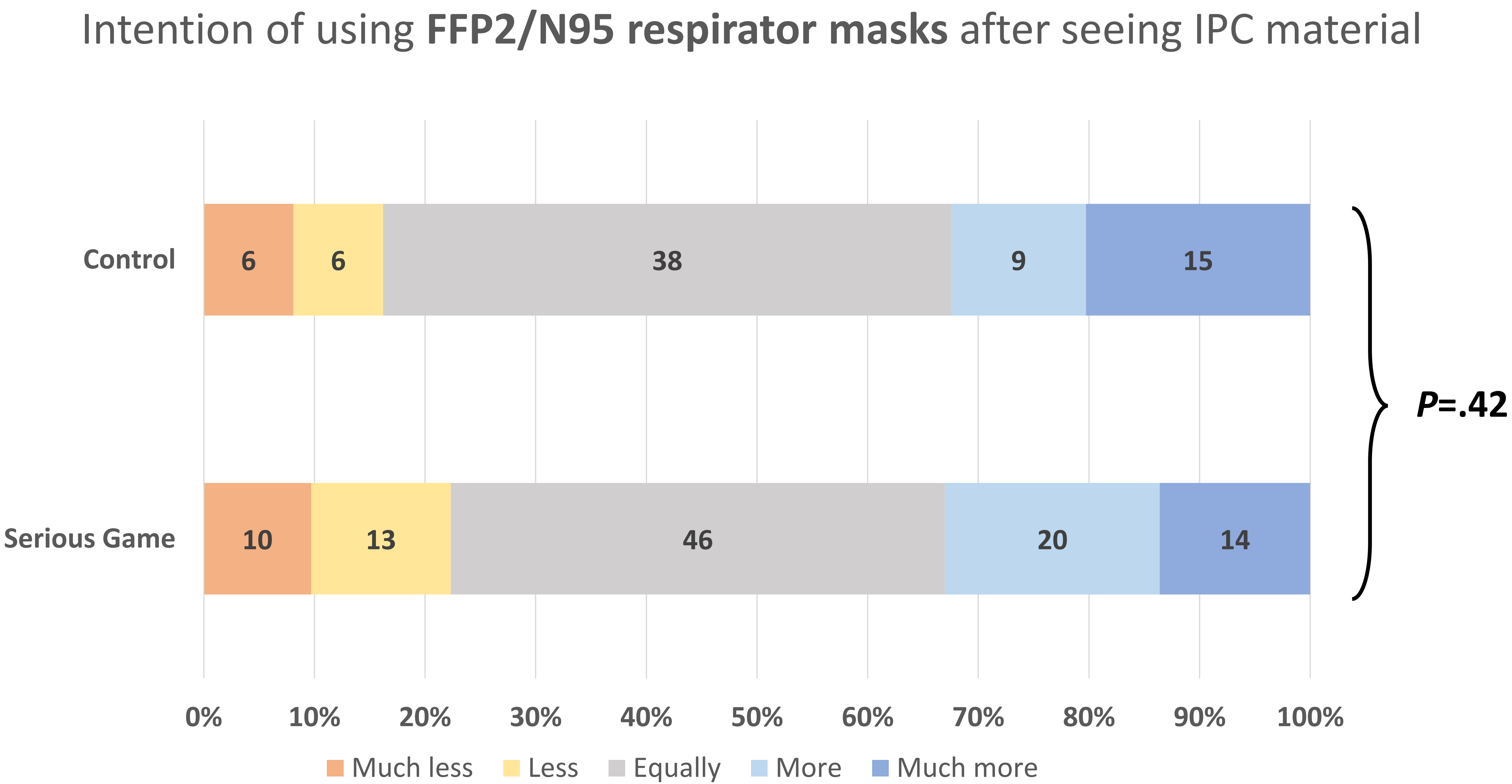

IPC: Infection Prevention and Control

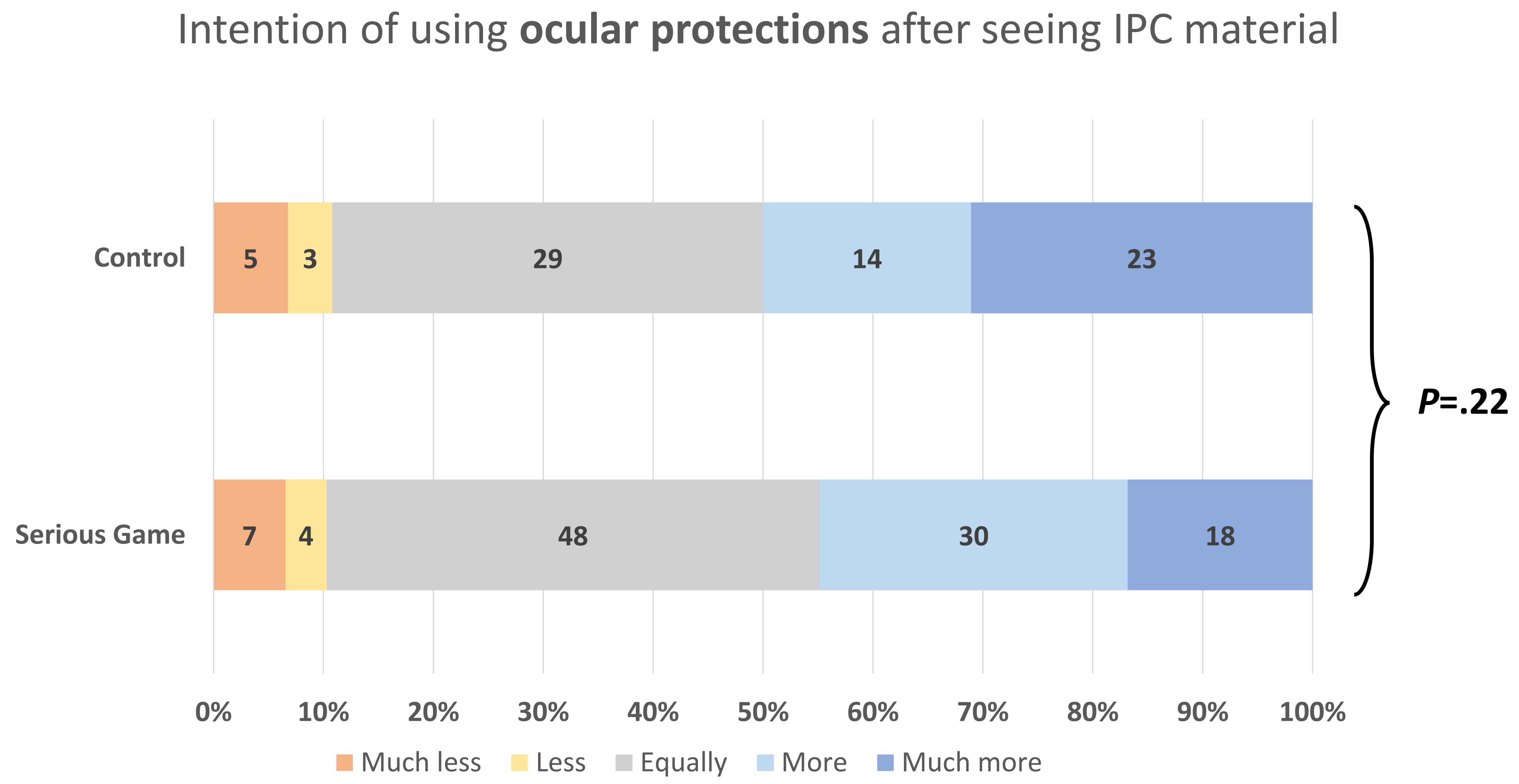

IPC: Infection Prevention and Control

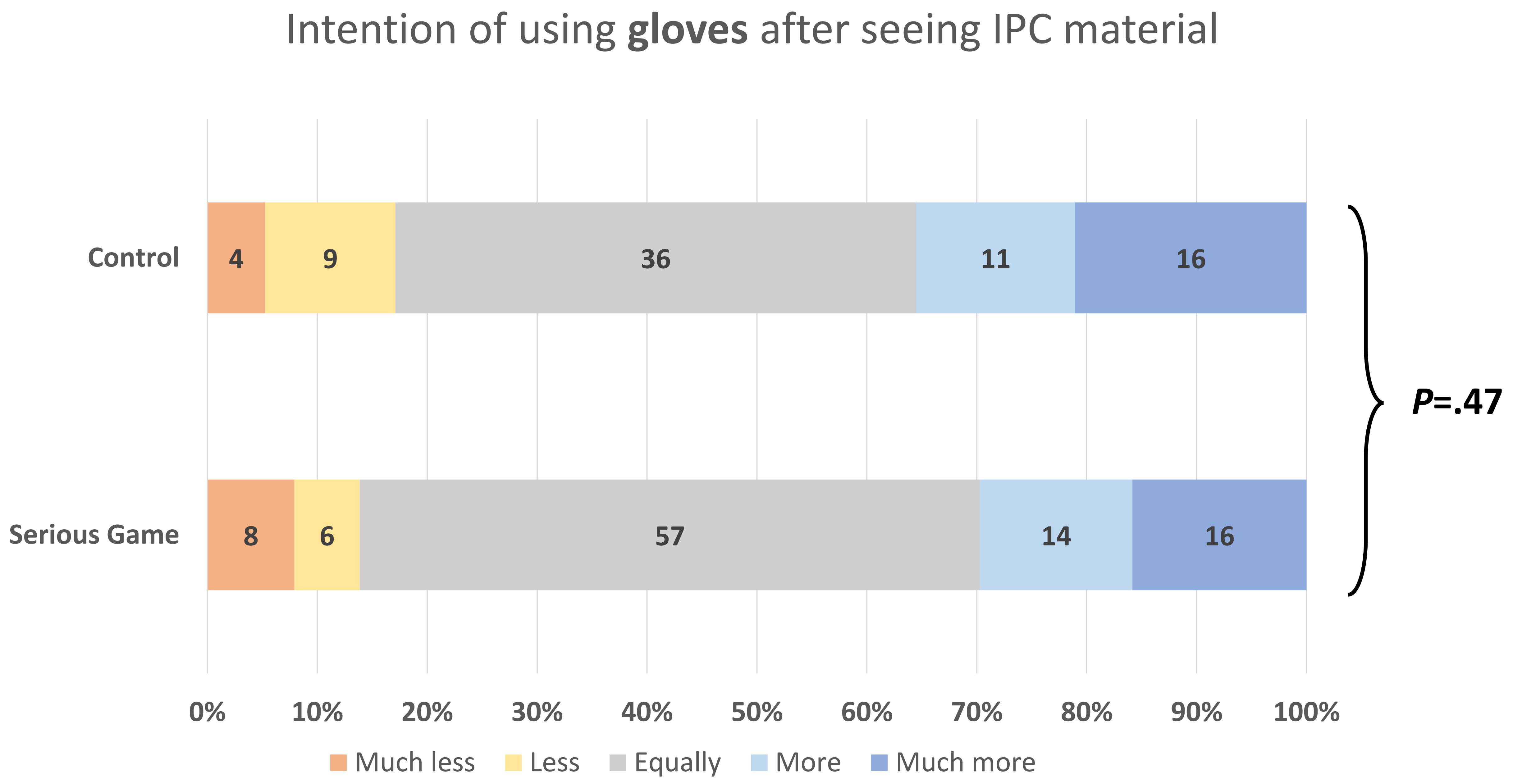

IPC: Infection Prevention and Control
