# Supplementary material for: Impact of a Serious Game (Escape COVID-19) on the Intention to Change COVID-19 Control Practices Among Employees of Long-term Care Facilities: Web-Based Randomized Controlled Trial
Source: J Med Internet Res. 2021 Mar 25;23(3):e27443. doi: 10.2196/27443 (PMC7996198; doi:10.2196/27443)
Supplement: Multimedia Appendix 8 [file jmir_v23i3e27443_app8.pdf]

CONSORT-EHEALTH (V 1.6.1) - Submission/Publication Form

The CONSORT-EHEALTH checklist is intended for authors of randomized trials evaluating web-based and Internet-based applications/interventions, including mobile interventions, electronic games (incl multiplayer games), social media, certain telehealth applications, and other interactive and/or networked electronic applications. Some of the items (e.g. all subitems under item 5 - description of the intervention) may also be applicable for other study designs.

The goal of the CONSORT EHEALTH checklist and guideline is to be
a) a guide for reporting for authors of RCTs,
b) to form a basis for appraisal of an ehealth trial (in terms of validity)

CONSORT-EHEALTH items/subitems are MANDATORY reporting items for studies published in the Journal of Medical Internet Research and other journals / scientific societies endorsing the checklist.

Items numbered 1., 2., 3., 4a., 4b etc are original CONSORT or CONSORT-NPT (non-pharmacologic treatment) items.
Items with Roman numerals (i., ii, iii, iv etc.) are CONSORT-EHEALTH extensions/clarifications.

As the CONSORT-EHEALTH checklist is still considered in a formative stage, we would ask that you also RATE ON A SCALE OF 1-5 how important/useful you feel each item is FOR THE PURPOSE OF THE CHECKLIST and reporting guideline (optional).

Mandatory reporting items are marked with a red *.
In the textboxes, either copy & paste the relevant sections from your manuscript into this form - please include any quotes from your manuscript in QUOTATION MARKS,
or answer directly by providing additional information not in the manuscript, or elaborating on why the item was not relevant for this study.

YOUR ANSWERS WILL BE PUBLISHED AS A SUPPLEMENTARY FILE TO YOUR PUBLICATION IN JMIR AND ARE CONSIDERED PART OF YOUR PUBLICATION (IF ACCEPTED).
Please fill in these questions diligently. Information will not be copyedited, so please use proper spelling and grammar, use correct capitalization, and avoid abbreviations.

DO NOT FORGET TO SAVE AS PDF _AND_ CLICK THE SUBMIT BUTTON SO YOUR ANSWERS ARE IN OUR DATABASE !!!

Citation Suggestion (if you append the pdf as Appendix we suggest to cite this paper in the caption):
Eysenbach G, CONSORT-EHEALTH Group
CONSORT-EHEALTH: Improving and Standardizing Evaluation Reports of Web-based and

Mobile Health Interventions
J Med Internet Res 2011;13(4):e126
URL: http://www.jmir.org/2011/4/e126/
doi: 10.2196/jmir.1923
PMID: 22209829

* Required

Your name *

First Last

Laurent Suppan

Primary Affiliation (short), City, Country *

University of Toronto, Toronto, Canada

University of Geneva Hospitals and Faculty of [illegible]

Your e-mail address *

abc@gmail.com

laurent.suppan@hcuge.ch

Title of your manuscript *

Provide the (draft) title of your manuscript.

Impact of a Serious Game, "Escape COVID-19", on the Intention to Change COVID-19 Control Practices among Employees of Long-term Care Facilities: Web-Based, Randomized Controlled Trial

Name of your App/Software/Intervention *

If there is a short and a long/alternate name, write the short name first and add the long name in brackets.

Escape COVID-19

Evaluated Version (if any)

e.g. "V1", "Release 2017-03-01", "Version 2.0.27913"

2.1.1

Language(s) *

What language is the intervention/app in? If multiple languages are available, separate by comma (e.g. "English, French")

French, English

URL of your Intervention Website or App

e.g. a direct link to the mobile app on app in appstore (itunes, Google Play), or URL of the website. If the intervention is a DVD or hardware, you can also link to an Amazon page.

https://escape-covid19.ch/

URL of an image/screenshot (optional)

https://asset.jmir.pub/assets/accb774981b4e4dc9747728f7406c554.png ; https://games.jm

Accessibility *

Can an enduser access the intervention presently?

- (selected) access is free and open
- access only for special usergroups, not open
- access is open to everyone, but requires payment/subscription/in-app purchases
- app/intervention no longer accessible
- Other:

Primary Medical Indication/Disease/Condition *

e.g. "Stress", "Diabetes", or define the target group in brackets after the condition, e.g. "Autism (Parents of children with)", "Alzheimers (Informal Caregivers of)"

COVID-19 infection prevention and control (IP[illegible]

Primary Outcomes measured in trial *

comma-separated list of primary outcomes reported in the trial

Proportion of long-term care facilities (LTCF) e[illegible]

Secondary/other outcomes

Are there any other outcomes the intervention is expected to affect?

Identification of factors associated with participant willingness to change their behavior, the reasons given by participants opposed to changing theirs, the potential motivators which could have led them to change, attrition, the IPC domains affected in participants who answered they were willing to change their IPC behavior, whether these participants would modify their use of specific personal protective equipment (PPE) items

Recommended "Dose" *

What do the instructions for users say on how often the app should be used?

- ○ Approximately Daily
- ○ Approximately Weekly
- ○ Approximately Monthly
- ○ Approximately Yearly
- ○ "as needed"
- ◉ Other: Not applicable / "single shot", but replayable

Approx. Percentage of Users (starters) still using the app as recommended after 3 months *

- ( ) unknown / not evaluated
- ( ) 0-10%
- ( ) 11-20%
- ( ) 21-30%
- ( ) 31-40%
- ( ) 41-50%
- ( ) 51-60%
- ( ) 61-70%
- ( ) 71%-80%
- ( ) 81-90%
- ( ) 91-100%
- (x) Other: Not applicable (see answer above)

Overall, was the app/intervention effective? *

- (x) yes: all primary outcomes were significantly better in intervention group vs control
- ( ) partly: SOME primary outcomes were significantly better in intervention group vs control
- ( ) no statistically significant difference between control and intervention
- ( ) potentially harmful: control was significantly better than intervention in one or more outcomes
- ( ) inconclusive: more research is needed
- ( ) Other:

Article Preparation Status/Stage *

At which stage in your article preparation are you currently (at the time you fill in this form)

- ○ not submitted yet - in early draft status
- ○ not submitted yet - in late draft status, just before submission
- ● submitted to a journal but not reviewed yet
- ○ submitted to a journal and after receiving initial reviewer comments
- ○ submitted to a journal and accepted, but not published yet
- ○ published
- ○ Other:

Journal *

If you already know where you will submit this paper (or if it is already submitted), please provide the journal name (if it is not JMIR, provide the journal name under "other")

- ○ not submitted yet / unclear where I will submit this
- ● Journal of Medical Internet Research (JMIR)
- ○ JMIR mHealth and UHealth
- ○ JMIR Serious Games
- ○ JMIR Mental Health
- ○ JMIR Public Health
- ○ JMIR Formative Research
- ○ Other JMIR sister journal
- ○ Other:

Is this a full powered effectiveness trial or a pilot/feasibility trial? *

- ( ) Pilot/feasibility
- (•) Fully powered

Manuscript tracking number *

If this is a JMIR submission, please provide the manuscript tracking number under "other" (The ms tracking number can be found in the submission acknowledgement email, or when you login as author in JMIR. If the paper is already published in JMIR, then the ms tracking number is the four-digit number at the end of the DOI, to be found at the bottom of each published article in JMIR)

- ( ) no ms number (yet) / not (yet) submitted to / published in JMIR
- (•) Other: 27443

TITLE AND ABSTRACT

1a) TITLE: Identification as a randomized trial in the title

1a) Does your paper address CONSORT item 1a? *

I.e does the title contain the phrase "Randomized Controlled Trial"? (if not, explain the reason under "other")

- (•) yes
- ( ) Other:

1a-i) Identify the mode of delivery in the title

Identify the mode of delivery. Preferably use “web-based” and/or “mobile” and/or “electronic game” in the title. Avoid ambiguous terms like “online”, “virtual”, “interactive”. Use “Internet-based” only if Intervention includes non-web-based Internet components (e.g. email), use “computer-based” or “electronic” only if offline products are used. Use “virtual” only in the context of “virtual reality” (3-D worlds). Use “online” only in the context of “online support groups”. Complement or substitute product names with broader terms for the class of products (such as “mobile” or “smart phone” instead of “iphone”), especially if the application runs on different platforms.

	1	2	3	4	5	
subitem not at all important	○	○	○	○	◉	essential

Clear selection

Does your paper address subitem 1a-i? *

Copy and paste relevant sections from manuscript title (include quotes in quotation marks "like this" to indicate direct quotes from your manuscript), or elaborate on this item by providing additional information not in the ms, or briefly explain why the item is not applicable/relevant for your study

"Web-Based" is included in the title: "Impact of a Serious Game, “Escape COVID-19”, on the Intention to Change COVID-19 Control Practices among Employees of Long-term Care Facilities: Web-Based, Randomized Controlled Trial"

1a-ii) Non-web-based components or important co-interventions in title

Mention non-web-based components or important co-interventions in title, if any (e.g., “with telephone support”).

	1	2	3	4	5	
subitem not at all important	○	○	○	◉	○	essential

Clear selection

Does your paper address subitem 1a-ii?

Copy and paste relevant sections from manuscript title (include quotes in quotation marks "like this" to indicate direct quotes from your manuscript), or elaborate on this item by providing additional information not in the ms, or briefly explain why the item is not applicable/relevant for your study

Indirectly: there were no other intervention than the web-based one. Therefore, no other intervention is mentioned in the title.

1a-iii) Primary condition or target group in the title

Mention primary condition or target group in the title, if any (e.g., “for children with Type I Diabetes”) Example: A Web-based and Mobile Intervention with Telephone Support for Children with Type I Diabetes: Randomized Controlled Trial

	1	2	3	4	5	
subitem not at all important	○	○	○	○	◉	essential

Clear selection

Does your paper address subitem 1a-iii? *

Copy and paste relevant sections from manuscript title (include quotes in quotation marks "like this" to indicate direct quotes from your manuscript), or elaborate on this item by providing additional information not in the ms, or briefly explain why the item is not applicable/relevant for your study

Yes, we targeted LTCF employees. This is explicitly mentioned in the title: "Impact of a Serious Game, “Escape COVID-19”, on the Intention to Change COVID-19 Control Practices among Employees of Long-term Care Facilities: Web-Based, Randomized Controlled Trial"

1b) ABSTRACT: Structured summary of trial design, methods, results, and conclusions

NPT extension: Description of experimental treatment, comparator, care providers, centers, and blinding status.

1b-i) Key features/functionalities/components of the intervention and comparator in the METHODS section of the ABSTRACT

Mention key features/functionalities/components of the intervention and comparator in the abstract. If possible, also mention theories and principles used for designing the site. Keep in mind the needs of systematic reviewers and indexers by including important synonyms. (Note: Only report in the abstract what the main paper is reporting. If this information is missing from the main body of text, consider adding it)

	1	2	3	4	5	
subitem not at all important	○	○	○	○	◉	essential

Clear selection

Does your paper address subitem 1b-i? *

Copy and paste relevant sections from the manuscript abstract (include quotes in quotation marks "like this" to indicate direct quotes from your manuscript), or elaborate on this item by providing additional information not in the ms, or briefly explain why the item is not applicable/relevant for your study

Yes: "In the control group, participants filled in a first questionnaire designed to gather demographic data and assess baseline knowledge before accessing regular online IPC guidelines." and "Conversely, the serious game group played "Escape COVID-19" after answering the first questionnaire but before answering the second one."

1b-ii) Level of human involvement in the METHODS section of the ABSTRACT

Clarify the level of human involvement in the abstract, e.g., use phrases like “fully automated” vs. “therapist/nurse/care provider/physician-assisted” (mention number and expertise of providers involved, if any). (Note: Only report in the abstract what the main paper is reporting. If this information is missing from the main body of text, consider adding it)

	1	2	3	4	5	
subitem not at all important	○	○	○	◉	○	essential

Clear selection

Does your paper address subitem 1b-ii?

Copy and paste relevant sections from the manuscript abstract (include quotes in quotation marks "like this" to indicate direct quotes from your manuscript), or elaborate on this item by providing additional information not in the ms, or briefly explain why the item is not applicable/relevant for your study

This is both implicit ("[...] forward information regarding the study and the connection procedure [...]") and explicit ("This was a web-based [...]"), but the exact words “fully automated” were not used in the abstract.

1b-iii) Open vs. closed, web-based (self-assessment) vs. face-to-face assessments in the METHODS section of the ABSTRACT

Mention how participants were recruited (online vs. offline), e.g., from an open access website or from a clinic or a closed online user group (closed usergroup trial), and clarify if this was a purely web-based trial, or there were face-to-face components (as part of the intervention or for assessment). Clearly say if outcomes were self-assessed through questionnaires (as common in web-based trials). Note: In traditional offline trials, an open trial (open-label trial) is a type of clinical trial in which both the researchers and participants know which treatment is being administered. To avoid confusion, use “blinded” or “unblinded” to indicated the level of blinding instead of “open”, as “open” in web-based trials usually refers to “open access” (i.e. participants can self-enrol). (Note: Only report in the abstract what the main paper is reporting. If this information is missing from the main body of text, consider adding it)

	1	2	3	4	5	
subitem not at all important	○	○	○	○	◉	essential

Clear selection

Does your paper address subitem 1b-iii?

Copy and paste relevant sections from the manuscript abstract (include quotes in quotation marks "like this" to indicate direct quotes from your manuscript), or elaborate on this item by providing additional information not in the ms, or briefly explain why the item is not applicable/relevant for your study

Yes: "The health authorities of Geneva (Switzerland) asked the managers of all LTCFs under their jurisdiction to forward information regarding the study and the connection procedure to all their employees, regardless of their professional status." and "In the control group, participants filled in a first questionnaire designed to gather demographic data and assess baseline knowledge before accessing regular online IPC guidelines. They then answered a second questionnaire which assessed their willingness to change their IPC practices and identify the reasons underlying their decision."

1b-iv) RESULTS section in abstract must contain use data

Report number of participants enrolled/assessed in each group, the use/uptake of the intervention (e.g., attrition/adherence metrics, use over time, number of logins etc.), in addition to primary/secondary outcomes. (Note: Only report in the abstract what the main paper is reporting. If this information is missing from the main body of text, consider adding it)

	1	2	3	4	5	
subitem not at all important	○	○	○	○	◉	essential

Clear selection

Does your paper address subitem 1b-iv?

Copy and paste relevant sections from the manuscript abstract (include quotes in quotation marks "like this" to indicate direct quotes from your manuscript), or elaborate on this item by providing additional information not in the ms, or briefly explain why the item is not applicable/relevant for your study

Yes: "A total of 295 answer sets were analyzed. The willingness to change behavior was higher in the serious game group (82% [119/145] vs 56% [84/150], P < .001)": therefore, there is both the whole number of answer sets (295) and the numbers in each group (145 in the serious game group, 150 in the control group).

1b-v) CONCLUSIONS/DISCUSSION in abstract for negative trials

Conclusions/Discussions in abstract for negative trials: Discuss the primary outcome - if the trial is negative (primary outcome not changed), and the intervention was not used, discuss whether negative results are attributable to lack of uptake and discuss reasons. (Note: Only report in the abstract what the main paper is reporting. If this information is missing from the main body of text, consider adding it)

	1	2	3	4	5	
subitem not at all important	○	○	○	○	◉	essential

Clear selection

Does your paper address subitem 1b-v?

Copy and paste relevant sections from the manuscript abstract (include quotes in quotation marks "like this" to indicate direct quotes from your manuscript), or elaborate on this item by providing additional information not in the ms, or briefly explain why the item is not applicable/relevant for your study

Not applicable: this was not a negative trial

INTRODUCTION

2a) In INTRODUCTION: Scientific background and explanation of rationale

2a-i) Problem and the type of system/solution

Describe the problem and the type of system/solution that is object of the study: intended as stand-alone intervention vs. incorporated in broader health care program? Intended for a particular patient population? Goals of the intervention, e.g., being more cost-effective to other interventions, replace or complement other solutions? (Note: Details about the intervention are provided in "Methods" under 5)

	1	2	3	4	5	
subitem not at all important	○	○	○	○	◉	essential

Clear selection

Does your paper address subitem 2a-i? *

Copy and paste relevant sections from the manuscript (include quotes in quotation marks "like this" to indicate direct quotes from your manuscript), or elaborate on this item by providing additional information not in the ms, or briefly explain why the item is not applicable/relevant for your study

Yes: "Long-term care facilities (LTCFs) have been hardly hit by the COVID-19 pandemic." and "This lack of resources can lead to suboptimal application of infection prevention and control (IPC) procedures, thus facilitating viral transmission." and "Moreover, in spite of well-established evidence regarding specific IPC practices such as hand hygiene and specific personal protective equipment (PPE) [19], most health care workers (HCW), including LTCF employees, only seldom apply them perfectly." and "To enhance the communication of appropriate IPC guidelines and improve their application by HCW and related staff who are regularly in contact with patients, we developed a web-based serious game called "Escape COVID-19" using Nicholson's RECIPE for meaningful gamification."

2a-ii) Scientific background, rationale: What is known about the (type of) system

Scientific background, rationale: What is known about the (type of) system that is the object of the study (be sure to discuss the use of similar systems for other conditions/diagnoses, if appropiate), motivation for the study, i.e. what are the reasons for and what is the context for this specific study, from which stakeholder viewpoint is the study performed, potential impact of findings [2]. Briefly justify the choice of the comparator.

	1	2	3	4	5	
subitem not at all important	○	○	○	○	◉	essential

Clear selection

Does your paper address subitem 2a-ii? *

Copy and paste relevant sections from the manuscript (include quotes in quotation marks "like this" to indicate direct quotes from your manuscript), or elaborate on this item by providing additional information not in the ms, or briefly explain why the item is not applicable/relevant for your study

Yes: ""While electronic learning (e-learning) interventions might be ineffective in teaching complex technical procedures, they are nevertheless useful in increasing knowledge and their use is generally associated with a high level of learner satisfaction. As the probability of executing an action is strongly linked to the intention of performing it according to the theory of planned behavior , an engaging serious game could enhance the dissemination of essential IPC guidelines and encourage LTCF employees to change their behavior regarding IPC practices."". Control materials are described in the METHODS: "[...] participants in the control group were shown a quick reminder of the most recent national guidelines published by the Federal Office of Public Health of the Swiss Confederation. They were also given links to local IPC guidelines for health care professionals (Vigigerme®) provided by the Geneva University Hospitals [...]."

2b) In INTRODUCTION: Specific objectives or hypotheses

Does your paper address CONSORT subitem 2b? *

Copy and paste relevant sections from the manuscript (include quotes in quotation marks "like this" to indicate direct quotes from your manuscript), or elaborate on this item by providing additional information not in the ms, or briefly explain why the item is not applicable/relevant for your study

Yes: "Our main objective was to assess the impact of “Escape COVID-19”, a web-based serious game, on the intention of LTCF employees to change their IPC practices. Our secondary objective was to determine the reasons underlying the potential willingness to change IPC practices or lack thereof."

METHODS

3a) Description of trial design (such as parallel, factorial) including allocation ratio

Does your paper address CONSORT subitem 3a? *

Copy and paste relevant sections from the manuscript (include quotes in quotation marks "like this" to indicate direct quotes from your manuscript), or elaborate on this item by providing additional information not in the ms, or briefly explain why the item is not applicable/relevant for your study

Yes: "This was a web-based, triple-blind (investigator, participants, data analyst), randomized controlled trial [...]".

3b) Important changes to methods after trial commencement (such as eligibility criteria), with reasons

Does your paper address CONSORT subitem 3b? *

Copy and paste relevant sections from the manuscript (include quotes in quotation marks "like this" to indicate direct quotes from your manuscript), or elaborate on this item by providing additional information not in the ms, or briefly explain why the item is not applicable/relevant for your study

Not applicable: no changes were made given the very limited timeframe ("which took place between November 5th and December 4th, 2020"). This was discussed in the protocol published in JMIR Research Protocols (https://www.researchprotocols.org/2020/12/e25595)

3b-i) Bug fixes, Downtimes, Content Changes

Bug fixes, Downtimes, Content Changes: ehealth systems are often dynamic systems. A description of changes to methods therefore also includes important changes made on the intervention or comparator during the trial (e.g., major bug fixes or changes in the functionality or content) (5-iii) and other “unexpected events” that may have influenced study design such as staff changes, system failures/downtimes, etc. [2].

	1	2	3	4	5	
subitem not at all important	○	○	○	○	◉	essential

Clear selection

Does your paper address subitem 3b-i?

Copy and paste relevant sections from the manuscript (include quotes in quotation marks "like this" to indicate direct quotes from your manuscript), or elaborate on this item by providing additional information not in the ms, or briefly explain why the item is not applicable/relevant for your study

There were no unexpected events, and no such event was expected given the limited timeframe (see answer above).

4a) Eligibility criteria for participants

Does your paper address CONSORT subitem 4a? *

Copy and paste relevant sections from the manuscript (include quotes in quotation marks "like this" to indicate direct quotes from your manuscript), or elaborate on this item by providing additional information not in the ms, or briefly explain why the item is not applicable/relevant for your study

Yes: "Regardless of their professional status, all LTCF employees working in Geneva, Switzerland, who received the invitation and elected to participate were included in this study and represented a convenience sample."

4a-i) Computer / Internet literacy

Computer / Internet literacy is often an implicit “de facto” eligibility criterion - this should be explicitly clarified.

	1	2	3	4	5	
subitem not at all important	○	○	○	○	◉	essential

Clear selection

Does your paper address subitem 4a-i?

Copy and paste relevant sections from the manuscript (include quotes in quotation marks "like this" to indicate direct quotes from your manuscript), or elaborate on this item by providing additional information not in the ms, or briefly explain why the item is not applicable/relevant for your study

Yes - given the setting and the population, this was included in the discussion: "Lack of eHealth literacy can probably hardly be blamed for the lack of participation. Indeed, LTCF employees increasingly use digital devices in the course of their work, and recent surveys have shown that eHealth literacy was rather high in HCW."

4a-ii) Open vs. closed, web-based vs. face-to-face assessments:

Open vs. closed, web-based vs. face-to-face assessments: Mention how participants were recruited (online vs. offline), e.g., from an open access website or from a clinic, and clarify if this was a purely web-based trial, or there were face-to-face components (as part of the intervention or for assessment), i.e., to what degree got the study team to know the participant. In online-only trials, clarify if participants were quasi-anonymous and whether having multiple identities was possible or whether technical or logistical measures (e.g., cookies, email confirmation, phone calls) were used to detect/prevent these.

	1	2	3	4	5	
subitem not at all important	○	○	○	○	◉	essential

Clear selection

Does your paper address subitem 4a-ii? *

Copy and paste relevant sections from the manuscript (include quotes in quotation marks "like this" to indicate direct quotes from your manuscript), or elaborate on this item by providing additional information not in the ms, or briefly explain why the item is not applicable/relevant for your study

All these issues were addresses: "[...] we informed the representatives of the health care authorities that, for methodological and clinical relevance, all LTCF employees should be invited to participate regardless of their professional status. We therefore provided them with an email template describing the objective of this study, the target population, and giving information regarding data protection (Multimedia Appendix 1). This template was sent by the public health authorities to all LTCF managers following a regular LTCF coordination meeting during which the characteristics of the study and of the serious game were detailed." "[...] participants were only asked to enter their email address, but were not asked for any other personal information and were not required to give their first and last names." and "To avoid having LTCF employees registering under a wrong institution, LTCF-specific accreditations were created for each facility. The full list of accreditations was sent to the public health authorities, who were informed they were to adapt our email template accordingly for each LTCF."

4a-iii) Information giving during recruitment

Information given during recruitment. Specify how participants were briefed for recruitment and in the informed consent procedures (e.g., publish the informed consent documentation as appendix, see also item X26), as this information may have an effect on user self-selection, user expectation and may also bias results.

	1	2	3	4	5	
subitem not at all important	○	○	○	○	◉	essential

Clear selection

Does your paper address subitem 4a-iii?

Copy and paste relevant sections from the manuscript (include quotes in quotation marks "like this" to indicate direct quotes from your manuscript), or elaborate on this item by providing additional information not in the ms, or briefly explain why the item is not applicable/relevant for your study

Yes - "We therefore provided [the health care authorities] with an email template describing the objective of this study, the target population, and giving information regarding data protection (Multimedia Appendix 1). This template was sent by the public health authorities to all LTCF managers following a regular LTCF coordination meeting during which the characteristics of the study and of the serious game were detailed."

4b) Settings and locations where the data were collected

Does your paper address CONSORT subitem 4b? *

Copy and paste relevant sections from the manuscript (include quotes in quotation marks "like this" to indicate direct quotes from your manuscript), or elaborate on this item by providing additional information not in the ms, or briefly explain why the item is not applicable/relevant for your study

Yes: "Regardless of their professional status, all LTCF employees working in Geneva, Switzerland, [...]"

4b-i) Report if outcomes were (self-)assessed through online questionnaires

Clearly report if outcomes were (self-)assessed through online questionnaires (as common in web-based trials) or otherwise.

	1	2	3	4	5	
subitem not at all important	○	○	○	○	◉	essential

Clear selection

Does your paper address subitem 4b-i? *

Copy and paste relevant sections from the manuscript (include quotes in quotation marks "like this" to indicate direct quotes from your manuscript), or elaborate on this item by providing additional information not in the ms, or briefly explain why the item is not applicable/relevant for your study

Yes, this is implicit: "Upon login, they were redirected to the first questionnaire (Multimedia Appendix 3) [...]" "This first questionnaire (Multimedia Appendix 3) was designed to assess the participants' baseline knowledge and to gather demographic data. It was developed using Community Surveys Pro (Corejoomla), which enables a completeness check and allows for the use of branching logic." and "This ensured that this questionnaire could only be answered by participants who had actually completed the game." and "Data was exported by LSu in Microsoft Excel (XLSX) and in comma-separated value (CSV) formats depending on the components before being imported, appended and merged under Stata (StataCorp LLC)."

4b-ii) Report how institutional affiliations are displayed

Report how institutional affiliations are displayed to potential participants [on ehealth media], as affiliations with prestigious hospitals or universities may affect volunteer rates, use, and reactions with regards to an intervention.(Not a required item – describe only if this may bias results)

	1	2	3	4	5	
subitem not at all important	○	○	○	○	◉	essential

Clear selection

Does your paper address subitem 4b-ii?

Copy and paste relevant sections from the manuscript (include quotes in quotation marks "like this" to indicate direct quotes from your manuscript), or elaborate on this item by providing additional information not in the ms, or briefly explain why the item is not applicable/relevant for your study

This is explicit in the screen captures provided (HUG - "Hôpitaux Universitaires de Genève" logo clearly displayed). The potential effect of the authoring institution is discussed: "[...] a potential mistrust in health authorities, in the institution authoring the game [...]"

5) The interventions for each group with sufficient details to allow replication, including how and when they were actually administered

5-i) Mention names, credential, affiliations of the developers, sponsors, and owners

Mention names, credential, affiliations of the developers, sponsors, and owners [6] (if authors/evaluators are owners or developer of the software, this needs to be declared in a "Conflict of interest" section or mentioned elsewhere in the manuscript).

	1	2	3	4	5	
subitem not at all important	○	○	○	○	●	essential

Clear selection

Does your paper address subitem 5-i?

Copy and paste relevant sections from the manuscript (include quotes in quotation marks "like this" to indicate direct quotes from your manuscript), or elaborate on this item by providing additional information not in the ms, or briefly explain why the item is not applicable/relevant for your study

Yes: "we developed a serious game called "Escape COVID-19" and, talking about the data analyst: "This investigator was not part of the serious game development team and did not co-author the original publication as we wanted to avoid any potential conflict of interest."

5-ii) Describe the history/development process

Describe the history/development process of the application and previous formative evaluations (e.g., focus groups, usability testing), as these will have an impact on adoption/use rates and help with interpreting results.

	1	2	3	4	5	
subitem not at all important	○	○	○	●	○	essential

Clear selection

Does your paper address subitem 5-ii?

Copy and paste relevant sections from the manuscript (include quotes in quotation marks "like this" to indicate direct quotes from your manuscript), or elaborate on this item by providing additional information not in the ms, or briefly explain why the item is not applicable/relevant for your study

Yes: "Briefly, “Escape COVID-19” was first developed in French under Articulate Storyline 3 (Articulate Global) through multiple iterations by using the first three steps of the SERES framework and Nicholson’s RECIPE for meaningful gamification. Graphical elements were designed by Eric Buche to lend a unique aspect to the game (Figures 6-7)." However, the development is described more precisely in another publication: https://games.jmir.org/2020/4/e24986

5-iii) Revisions and updating

Revisions and updating. Clearly mention the date and/or version number of the application/intervention (and comparator, if applicable) evaluated, or describe whether the intervention underwent major changes during the evaluation process, or whether the development and/or content was “frozen” during the trial. Describe dynamic components such as news feeds or changing content which may have an impact on the replicability of the intervention (for unexpected events see item 3b).

	1	2	3	4	5	
subitem not at all important	○	○	○	○	◉	essential

Clear selection

Does your paper address subitem 5-iii?

Copy and paste relevant sections from the manuscript (include quotes in quotation marks "like this" to indicate direct quotes from your manuscript), or elaborate on this item by providing additional information not in the ms, or briefly explain why the item is not applicable/relevant for your study

This was dealt with in more details in the protocol (https://www.researchprotocols.org/2020/12/e25595) - however, the version is clearly mentioned ("[...] “Escape COVID-19” (version 2.1.1) was launched once participants in the serious game group had completed the first questionnaire. ") and the limited timeframe should help lift any doubt ("This was a web-based, triple-blind, randomized controlled trial, which took place between November 5th and December 4th, 2020.")

5-iv) Quality assurance methods

Provide information on quality assurance methods to ensure accuracy and quality of information provided [1], if applicable.

	1	2	3	4	5	
subitem not at all important	○	○	○	○	◉	essential

Clear selection

Does your paper address subitem 5-iv?

Copy and paste relevant sections from the manuscript (include quotes in quotation marks "like this" to indicate direct quotes from your manuscript), or elaborate on this item by providing additional information not in the ms, or briefly explain why the item is not applicable/relevant for your study

Yes: "It was developed using Community Surveys Pro (Corejoomla) [44], which enables a completeness check [...]" and "The questions could not be changed once a page had been completed." and "Daily backups were performed throughout the study period. They were triggered by a cron job script and uploaded on a physically separate server through an encrypted connection."

5-v) Ensure replicability by publishing the source code, and/or providing screenshots/screen-capture video, and/or providing flowcharts of the algorithms used

Ensure replicability by publishing the source code, and/or providing screenshots/screen-capture video, and/or providing flowcharts of the algorithms used. Replicability (i.e., other researchers should in principle be able to replicate the study) is a hallmark of scientific reporting.

	1	2	3	4	5	
subitem not at all important	○	○	○	○	◉	essential

Clear selection

Does your paper address subitem 5-v?

Copy and paste relevant sections from the manuscript (include quotes in quotation marks "like this" to indicate direct quotes from your manuscript), or elaborate on this item by providing additional information not in the ms, or briefly explain why the item is not applicable/relevant for your study

This is essential: all questions are available in the Multimedia Appendices (and in the original protocol - https://www.researchprotocols.org/2020/12/e25595), and the serious game can be freely accessed on the internet (https://escape-covid19.ch/). Moreover, we have clearly stated that "The first or last authors can be contacted to obtain Shareable Content Object Reference Model (SCORM) packages, which can be reused freely for research and educational purposes."

5-vi) Digital preservation

Digital preservation: Provide the URL of the application, but as the intervention is likely to change or disappear over the course of the years; also make sure the intervention is archived (Internet Archive, webcitation.org, and/or publishing the source code or screenshots/videos alongside the article). As pages behind login screens cannot be archived, consider creating demo pages which are accessible without login.

	1	2	3	4	5	
subitem not at all important	○	○	○	○	●	essential

Clear selection

Does your paper address subitem 5-vi?

Copy and paste relevant sections from the manuscript (include quotes in quotation marks "like this" to indicate direct quotes from your manuscript), or elaborate on this item by providing additional information not in the ms, or briefly explain why the item is not applicable/relevant for your study

Alas, webcitation.org does not accept archiving requests anymore. The URL of the serious game is however provided (https://escape-covid19.ch/)

5-vii) Access

Access: Describe how participants accessed the application, in what setting/context, if they had to pay (or were paid) or not, whether they had to be a member of specific group. If known, describe how participants obtained “access to the platform and Internet” [1]. To ensure access for editors/reviewers/readers, consider to provide a “backdoor” login account or demo mode for reviewers/readers to explore the application (also important for archiving purposes, see vi).

	1	2	3	4	5	
subitem not at all important	○	○	○	○	◉	essential

Clear selection

Does your paper address subitem 5-vii? *

Copy and paste relevant sections from the manuscript (include quotes in quotation marks "like this" to indicate direct quotes from your manuscript), or elaborate on this item by providing additional information not in the ms, or briefly explain why the item is not applicable/relevant for your study

Yes - the game is freely available, so there is no need for a "backdoor", and our dataset has been uploaded to the Mendeley repository (doi: 10.17632/7cjmmntf6j.1), though under embargo. The curated DTA file used by the data analyst can be obtained by contacting the last author.
Regarding access: "After clicking on the “Access” button, a registration form, identical for both groups, was shown (Figure 4). This form was created using Membership Pro (Joomdonation) as this component enabled us to allocate users to specific groups, to create specific fields, and to disable the “Name” field. Therefore, participants were only asked to enter their email address, but were not asked for any other personal information and were not required to give their first and last names."

5-viii) Mode of delivery, features/functionalities/components of the intervention and comparator, and the theoretical framework

Describe mode of delivery, features/functionalities/components of the intervention and comparator, and the theoretical framework [6] used to design them (instructional strategy [1], behaviour change techniques, persuasive features, etc., see e.g., [7, 8] for terminology). This includes an in-depth description of the content (including where it is coming from and who developed it) [1],” whether [and how] it is tailored to individual circumstances and allows users to track their progress and receive feedback” [6]. This also includes a description of communication delivery channels and – if computer-mediated communication is a component – whether communication was synchronous or asynchronous [6]. It also includes information on presentation strategies [1], including page design principles, average amount of text on pages, presence of hyperlinks to other resources, etc. [1].

	1	2	3	4	5	
subitem not at all important	○	○	○	○	◉	essential

Clear selection

Does your paper address subitem 5-viii? *

Copy and paste relevant sections from the manuscript (include quotes in quotation marks "like this" to indicate direct quotes from your manuscript), or elaborate on this item by providing additional information not in the ms, or briefly explain why the item is not applicable/relevant for your study

Yes - this is described throughout the manuscript.Just a few examples: "[...]“Escape COVID-19” was first developed in French under Articulate Storyline 3 (Articulate Global) through multiple iterations by using the first three steps of the SERES framework [23,51,52] and Nicholson’s RECIPE for meaningful gamification." and "As the probability of executing an action is strongly linked to the intention of performing it according to the theory of planned behavior, an engaging serious game could enhance the dissemination of essential IPC guidelines"

5-ix) Describe use parameters

Describe use parameters (e.g., intended "doses" and optimal timing for use). Clarify what instructions or recommendations were given to the user, e.g., regarding timing, frequency, heaviness of use, if any, or was the intervention used ad libitum.

	1	2	3	4	5	
subitem not at all important	○	○	○	●	○	essential

Clear selection

Does your paper address subitem 5-ix?

Copy and paste relevant sections from the manuscript (include quotes in quotation marks "like this" to indicate direct quotes from your manuscript), or elaborate on this item by providing additional information not in the ms, or briefly explain why the item is not applicable/relevant for your study

Not applicable, as this was a "single shot" serious game, though participants could re-access it at will.

5-x) Clarify the level of human involvement

Clarify the level of human involvement (care providers or health professionals, also technical assistance) in the e-intervention or as co-intervention (detail number and expertise of professionals involved, if any, as well as "type of assistance offered, the timing and frequency of the support, how it is initiated, and the medium by which the assistance is delivered". It may be necessary to distinguish between the level of human involvement required for the trial, and the level of human involvement required for a routine application outside of a RCT setting (discuss under item 21 – generalizability).

	1	2	3	4	5	
subitem not at all important	○	○	○	○	●	essential

Clear selection

Does your paper address subitem 5-x?

Copy and paste relevant sections from the manuscript (include quotes in quotation marks "like this" to indicate direct quotes from your manuscript), or elaborate on this item by providing additional information not in the ms, or briefly explain why the item is not applicable/relevant for your study

This is implicit throughout the Study Design and Settings subsection of the methods, and the only human involvement was in sending the emails: "Having been informed by IPC specialists from the Geneva University Hospitals of the development of "Escape COVID-19", the public health authorities of Geneva (Switzerland) were interested in giving access to this serious game to the employees of all LTCFs under their jurisdiction. They therefore provided us with a comprehensive list of all such facilities, but insisted on liaising themselves with LTCF managers. Because LTCF employees other than HCW can often be in contact with residents, we informed the representatives of the health care authorities that, for methodological and clinical relevance, all LTCF employees should be invited to participate regardless of their professional status. We therefore provided them with an email template describing the objective of this study, the target population, and giving information regarding data protection (Multimedia Appendix 1). This template was sent by the public health authorities to all LTCF managers following a regular LTCF coordination meeting during which the characteristics of the study and of the serious game were detailed." and "Stratification was achieved by having LTCF employees click on a link specific to their structure. When they clicked on this link, participants were automatically randomized to one of the two study paths by GegaByte's Random Article module (GegaByte technologies). This process was invisible to the end user and there was no way either participants or investigators could influence group allocation.
General information regarding the study, the need to register with a valid email address, and the need to allow the reception of emails coming from the website's domain was then displayed (Figure 3)."

5-xi) Report any prompts/reminders used

Report any prompts/reminders used: Clarify if there were prompts (letters, emails, phone calls, SMS) to use the application, what triggered them, frequency etc. It may be necessary to distinguish between the level of prompts/reminders required for the trial, and the level of prompts/reminders for a routine application outside of a RCT setting (discuss under item 21 – generalizability).

	1	2	3	4	5	
subitem not at all important	○	○	○	○	◉	essential

Clear selection

Does your paper address subitem 5-xi? *

Copy and paste relevant sections from the manuscript (include quotes in quotation marks "like this" to indicate direct quotes from your manuscript), or elaborate on this item by providing additional information not in the ms, or briefly explain why the item is not applicable/relevant for your study

Unfortunately, all communication had to go through the Geneva health care authorities ("They therefore provided us with a comprehensive list of all such facilities, but insisted on liaising themselves with LTCF managers"), who were also unable to communicate directly with the potential participants - this is a clear limitation and is mentioned as such in the discussion: "Regardless of the reason, the ability of health authorities to successfully convey critical messages by efficient vectors to LTCF employees should be assessed specifically to solve potential communication issues."

5-xii) Describe any co-interventions (incl. training/support)

Describe any co-interventions (incl. training/support): Clearly state any interventions that are provided in addition to the targeted eHealth intervention, as ehealth intervention may not be designed as stand-alone intervention. This includes training sessions and support [1]. It may be necessary to distinguish between the level of training required for the trial, and the level of training for a routine application outside of a RCT setting (discuss under item 21 – generalizability.

	1	2	3	4	5	
subitem not at all important	○	○	○	○	◉	essential

Clear selection

Does your paper address subitem 5-xii? *

Copy and paste relevant sections from the manuscript (include quotes in quotation marks "like this" to indicate direct quotes from your manuscript), or elaborate on this item by providing additional information not in the ms, or briefly explain why the item is not applicable/relevant for your study

Not applicable: given the design of this study, the use of this serious game was clearly a standalone intervention.

6a) Completely defined pre-specified primary and secondary outcome measures, including how and when they were assessed

Does your paper address CONSORT subitem 6a? *

Copy and paste relevant sections from the manuscript (include quotes in quotation marks "like this" to indicate direct quotes from your manuscript), or elaborate on this item by providing additional information not in the ms, or briefly explain why the item is not applicable/relevant for your study

Yes: "The primary outcome was the proportion of LTCF employees who answered they were willing to change their IPC practices after seeing either the serious game or the control material. The secondary outcomes were the identification of factors associated with participant willingness to change their behavior, the reasons given by participants opposed to changing theirs, and the potential motivators which could have led them to change. Attrition was evaluated at each stage of the study. We also assessed the IPC domains affected in participants who answered they were willing to change their IPC behavior, and whether these participants would modify their use of specific PPE items. Thirteen questions were used to assess these latter outcomes. Therefore, conversely to the previous outcomes, answering questions related to IPC domains and PPE items were not compulsory to limit attrition."

6a-i) Online questionnaires: describe if they were validated for online use and apply CHERRIES items to describe how the questionnaires were designed/deployed

If outcomes were obtained through online questionnaires, describe if they were validated for online use and apply CHERRIES items to describe how the questionnaires were designed/deployed [9].

	1	2	3	4	5	
subitem not at all important	○	○	○	○	◉	essential

Clear selection

Does your paper address subitem 6a-i?

Copy and paste relevant sections from manuscript text

Yes: "[...] incorporates relevant elements from the CHERRIES checklist [...] and "It was developed using Community Surveys Pro (Corejoomla) , which enables a completeness check and allows for the use of branching logic. The number of initial questions was kept at a minimum and branching logic was used to try to limit attrition . All multiple-choice and multiple-answer questions were mandatory and had to be answered before participants could move on to the next step. The questions could not be changed once a page had been completed."

6a-ii) Describe whether and how "use" (including intensity of use/dosage) was defined/measured/monitored

Describe whether and how "use" (including intensity of use/dosage) was defined/measured/monitored (logins, logfile analysis, etc.). Use/adoption metrics are important process outcomes that should be reported in any ehealth trial.

	1	2	3	4	5	
subitem not at all important	○	○	○	○	◉	essential

Clear selection

Does your paper address subitem 6a-ii?

Copy and paste relevant sections from manuscript text

Not applicable, as this was a single shot intervention. We however made sure that the participants (in the serious game group) had indeed played the game: "The only way participants could access the second questionnaire was by clicking on the "Terminer le jeu" (End the game) button"

6a-iii) Describe whether, how, and when qualitative feedback from participants was obtained

Describe whether, how, and when qualitative feedback from participants was obtained (e.g., through emails, feedback forms, interviews, focus groups).

	1	2	3	4	5	
subitem not at all important	○	○	○	○	◉	essential

Clear selection

Does your paper address subitem 6a-iii?

Copy and paste relevant sections from manuscript text

Yes - this was necessary to compute the primary outcome, and was achieved through the use of the second questionnaire ("[...] these participants were asked to complete the second questionnaire (Multimedia Appendix 4)."), which is available as a Multimedia Appendix

6b) Any changes to trial outcomes after the trial commenced, with reasons

Does your paper address CONSORT subitem 6b? *

Copy and paste relevant sections from the manuscript (include quotes in quotation marks "like this" to indicate direct quotes from your manuscript), or elaborate on this item by providing additional information not in the ms, or briefly explain why the item is not applicable/relevant for your study

This is implicit given the very limited timeframe ("[...] took place between November 5th and December 4th, 2020"), and was discussed in the protocol (https://www.researchprotocols.org/2020/12/e25595)

7a) How sample size was determined

NPT: When applicable, details of whether and how the clustering by care provides or centers was addressed

7a-i) Describe whether and how expected attrition was taken into account when calculating the sample size

Describe whether and how expected attrition was taken into account when calculating the sample size.

	1	2	3	4	5	
subitem not at all important	○	○	○	○	◉	essential

Clear selection

Does your paper address subitem 7a-i?

Copy and paste relevant sections from manuscript title (include quotes in quotation marks "like this" to indicate direct quotes from your manuscript), or elaborate on this item by providing additional information not in the ms, or briefly explain why the item is not applicable/relevant for your study

Yes, this was detailed in the protocol (https://www.researchprotocols.org/2020/12/e25595) and stated in the manuscript ("Regardless of their professional status, all LTCF employees working in Geneva, Switzerland, who received the invitation and elected to participate were included in this study and represented a convenience sample. As the number of eligible employees was estimated to near 4’000 people, we hoped for a participation rate of around 20%. This would have allowed us to detect a difference of 10% at the .05 significance level with a power of 80% as we had calculated that 388 participants would have been needed in each group to detect such a difference.")

7b) When applicable, explanation of any interim analyses and stopping guidelines

Does your paper address CONSORT subitem 7b? *

Copy and paste relevant sections from the manuscript (include quotes in quotation marks "like this" to indicate direct quotes from your manuscript), or elaborate on this item by providing additional information not in the ms, or briefly explain why the item is not applicable/relevant for your study

Not applicable (not planned for - cf. protocol - https://www.researchprotocols.org/2020/12/e25595)

8a) Method used to generate the random allocation sequence

NPT: When applicable, how care providers were allocated to each trial group

Does your paper address CONSORT subitem 8a? *

Copy and paste relevant sections from the manuscript (include quotes in quotation marks "like this" to indicate direct quotes from your manuscript), or elaborate on this item by providing additional information not in the ms, or briefly explain why the item is not applicable/relevant for your study

Yes: "When they clicked on this link, participants were automatically randomized to one of the two study paths by GegaByte's Random Article module (GegaByte technologies). This process was invisible to the end user and there was no way either participants or investigators could influence group allocation."

8b) Type of randomisation; details of any restriction (such as blocking and block size)

Does your paper address CONSORT subitem 8b? *

Copy and paste relevant sections from the manuscript (include quotes in quotation marks "like this" to indicate direct quotes from your manuscript), or elaborate on this item by providing additional information not in the ms, or briefly explain why the item is not applicable/relevant for your study

Yes: "When they clicked on this link, participants were automatically randomized to one of the two study paths by GegaByte's Random Article module (GegaByte technologies). This process was invisible to the end user and there was no way either participants or investigators could influence group allocation."

9) Mechanism used to implement the random allocation sequence (such as sequentially numbered containers), describing any steps taken to conceal the sequence until interventions were assigned

Does your paper address CONSORT subitem 9? *

Copy and paste relevant sections from the manuscript (include quotes in quotation marks "like this" to indicate direct quotes from your manuscript), or elaborate on this item by providing additional information not in the ms, or briefly explain why the item is not applicable/relevant for your study

Yes: "When they clicked on this link, participants were automatically randomized to one of the two study paths by GegaByte's Random Article module (GegaByte technologies). This process was invisible to the end user and there was no way either participants or investigators could influence group allocation."

10) Who generated the random allocation sequence, who enrolled participants, and who assigned participants to interventions

Does your paper address CONSORT subitem 10? *

Copy and paste relevant sections from the manuscript (include quotes in quotation marks "like this" to indicate direct quotes from your manuscript), or elaborate on this item by providing additional information not in the ms, or briefly explain why the item is not applicable/relevant for your study

Yes:
Enrollment: "[...] we informed the representatives of the health care authorities that, for methodological and clinical relevance, all LTCF employees should be invited to participate regardless of their professional status. We therefore provided them with an email template describing the objective of this study, the target population, and giving information regarding data protection (Multimedia Appendix 1). This template was sent by the public health authorities to all LTCF managers following a regular LTCF coordination meeting during which the characteristics of the study and of the serious game were detailed." and "Regardless of their professional status, all LTCF employees working in Geneva, Switzerland, who received the invitation and elected to participate were included in this study and represented a convenience sample."
Randomization / allocation: "When they clicked on this link, participants were automatically randomized to one of the two study paths by GegaByte's Random Article module (GegaByte technologies). This process was invisible to the end user and there was no way either participants or investigators could influence group allocation."

11a) If done, who was blinded after assignment to interventions (for example, participants, care providers, those assessing outcomes) and how

NPT: Whether or not administering co-interventions were blinded to group assignment

11a-i) Specify who was blinded, and who wasn't

Specify who was blinded, and who wasn't. Usually, in web-based trials it is not possible to blind the participants [1, 3] (this should be clearly acknowledged), but it may be possible to blind outcome assessors, those doing data analysis or those administering co-interventions (if any).

	1	2	3	4	5	
subitem not at all important	○	○	○	○	◉	essential

Clear selection

Does your paper address subitem 11a-i? *

Copy and paste relevant sections from the manuscript (include quotes in quotation marks "like this" to indicate direct quotes from your manuscript), or elaborate on this item by providing additional information not in the ms, or briefly explain why the item is not applicable/relevant for your study

Participants: "Participants, including LTCF managers, were unaware that they would be allocated to one of two different study arms"
Investigators: "When they clicked on this link, participants were automatically randomized to one of the two study paths by GegaByte's Random Article module (GegaByte technologies). This process was invisible to the end user and there was no way either participants or investigators could influence group allocation."
Data analyst: "Data was exported by LSu in Microsoft Excel (XLSX) and in comma-separated value (CSV) formats depending on the components before being imported, appended and merged under Stata (StataCorp LLC). The groups were renamed using neutral names ("Atreides" and "Corrino"), the fields which could have led to an unblinding of the data analyst removed, and the curated DTA file transmitted to LSt who used Stata 15.1 for statistical analysis. "

11a-ii) Discuss e.g., whether participants knew which intervention was the “intervention of interest” and which one was the “comparator”

Informed consent procedures (4a-ii) can create biases and certain expectations - discuss e.g., whether participants knew which intervention was the “intervention of interest” and which one was the “comparator”.

	1	2	3	4	5	
subitem not at all important	○	○	○	○	●	essential

Clear selection

Does your paper address subitem 11a-ii?

Copy and paste relevant sections from the manuscript (include quotes in quotation marks "like this" to indicate direct quotes from your manuscript), or elaborate on this item by providing additional information not in the ms, or briefly explain why the item is not applicable/relevant for your study

Yes - "Participants, including LTCF managers, were unaware that they would be allocated to one of two different study arms"

11b) If relevant, description of the similarity of interventions

(this item is usually not relevant for ehealth trials as it refers to similarity of a placebo or sham intervention to a active medication/intervention)

Does your paper address CONSORT subitem 11b? *

Copy and paste relevant sections from the manuscript (include quotes in quotation marks "like this" to indicate direct quotes from your manuscript), or elaborate on this item by providing additional information not in the ms, or briefly explain why the item is not applicable/relevant for your study

Yes - in the discussion: "As the serious game contained little material other than that presented in the standard IPC guidelines"

12a) Statistical methods used to compare groups for primary and secondary outcomes

NPT: When applicable, details of whether and how the clustering by care providers or centers was addressed

Does your paper address CONSORT subitem 12a? *

Copy and paste relevant sections from the manuscript (include quotes in quotation marks "like this" to indicate direct quotes from your manuscript), or elaborate on this item by providing additional information not in the ms, or briefly explain why the item is not applicable/relevant for your study

Yes: "Univariable and multivariable logistic regression were used to assess the primary outcome, with adjustment performed according to prior knowledge (expressed as the percentage of correct answers), professional status, and facility. The log-linearity assumption was checked graphically and the goodness-of-fit was tested using the Hosmer-Lemeshow test. As randomization was stratified by center, we adjusted for this in the analysis by employing a random effects logistic regression model, using LTCF as a random effect. We tested the null hypothesis of absence of random effect using a chi-bar-squared test. We calculated the intraclass correlation coefficient (ICC) to quantify to what extent responses in a single LTCF were correlated.
Secondary outcomes were analyzed by assigning numerical values to the answers gathered through the use of Likert scales. As the domains potentially affected by a change in behavior were assessed using Likert scales ranging from 1 (not at all) to 6 (very much), the same values (i.e., a score ranging from 1 to 6) were assigned to each item. The composite outcome was the sum of these 9 questions and was analyzed through univariable linear regression then adjusted by employing a mixed effects model, using LTCF as a random effect and same adjustment variables as for the primary outcome.
The changes in the use of specific PPE items were assessed using 5-point Likert scales, ranging from “much less” to “much more”. An odd number was decided upon to allow participants to give a neutral answer. Values ranging from -2 to +2 were therefore assigned to each answer, with positive values attributed to changes enhancing IPC behavior. A composite outcome was generated by summing up these values. We used the same statistical method as for the prior composite outcome. When computing composite outcomes, the same weight was applied to all questions. As a reduction in the use of N95 respirators can also be considered as desirable depending on the setting, a sensitivity analysis was performed by analyzing the composite outcome with and without this particular item. Each individual question based on a Likert scale was also analyzed separately and the results are presented graphically, either in the manuscript or in Multimedia Appendices. No imputation technique was used."

12a-i) Imputation techniques to deal with attrition / missing values

Imputation techniques to deal with attrition / missing values: Not all participants will use the intervention/comparator as intended and attrition is typically high in ehealth trials. Specify how participants who did not use the application or dropped out from the trial were treated in the statistical analysis (a complete case analysis is strongly discouraged, and simple imputation techniques such as LOCF may also be problematic [4]).

	1	2	3	4	5	
subitem not at all important	○	○	○	○	◉	essential

Clear selection

Does your paper address subitem 12a-i? *

Copy and paste relevant sections from the manuscript (include quotes in quotation marks "like this" to indicate direct quotes from your manuscript), or elaborate on this item by providing additional information not in the ms, or briefly explain why the item is not applicable/relevant for your study

Yes: "No imputation technique was used."

12b) Methods for additional analyses, such as subgroup analyses and adjusted analyses

Does your paper address CONSORT subitem 12b? *

Copy and paste relevant sections from the manuscript (include quotes in quotation marks "like this" to indicate direct quotes from your manuscript), or elaborate on this item by providing additional information not in the ms, or briefly explain why the item is not applicable/relevant for your study

Yes: "As randomization was stratified by center, we adjusted for this in the analysis by employing a random effects logistic regression model, using LTCF as a random effect. We tested the null hypothesis of absence of random effect using a chi-bar-squared test. We calculated the intraclass correlation coefficient (ICC) to quantify to what extent responses in a single LTCF were correlated."

X26) REB/IRB Approval and Ethical Considerations [recommended as subheading under "Methods"] (not a CONSORT item)

X26-i) Comment on ethics committee approval

	1	2	3	4	5	
subitem not at all important	○	○	○	○	◉	essential

Clear selection

Does your paper address subitem X26-i?

Copy and paste relevant sections from the manuscript (include quotes in quotation marks "like this" to indicate direct quotes from your manuscript), or elaborate on this item by providing additional information not in the ms, or briefly explain why the item is not applicable/relevant for your study

Yes: "A declaration of no objection was issued by our regional ethics committee (Req-2020-01262) as such projects do not fall within the scope of the Swiss federal law on human research."

x26-ii) Outline informed consent procedures

Outline informed consent procedures e.g., if consent was obtained offline or online (how? Checkbox, etc.?), and what information was provided (see 4a-ii). See [6] for some items to be included in informed consent documents.

	1	2	3	4	5	
subitem not at all important	○	○	○	○	◉	essential

Clear selection

Does your paper address subitem X26-ii?

Copy and paste relevant sections from the manuscript (include quotes in quotation marks "like this" to indicate direct quotes from your manuscript), or elaborate on this item by providing additional information not in the ms, or briefly explain why the item is not applicable/relevant for your study

Yes: "Participants were informed that clicking on the activation link was considered as consent to participate in the study."

X26-iii) Safety and security procedures

Safety and security procedures, incl. privacy considerations, and any steps taken to reduce the likelihood or detection of harm (e.g., education and training, availability of a hotline)

	1	2	3	4	5	
subitem not at all important	○	○	○	○	◉	essential

Clear selection

Does your paper address subitem X26-iii?

Copy and paste relevant sections from the manuscript (include quotes in quotation marks "like this" to indicate direct quotes from your manuscript), or elaborate on this item by providing additional information not in the ms, or briefly explain why the item is not applicable/relevant for your study

Yes: "This email also contained a reminder regarding data handling and security (Multimedia Appendix 2)." and "Only two authors (MS and LSu) had access to the administration console and to the data, which was stored on an encrypted MySQL compatible database (MariaDB 5.5.5, MariaDB Foundation). Daily backups were performed throughout the study period. They were triggered by a cron job script and uploaded on a physically separate server through an encrypted connection." There is even more detailed information regarding the hosting and the security components in the protocol (https://www.researchprotocols.org/2020/12/e25595).

RESULTS

13a) For each group, the numbers of participants who were randomly assigned, received intended treatment, and were analysed for the primary outcome

NPT: The number of care providers or centers performing the intervention in each group and the number of patients treated by each care provider in each center

Does your paper address CONSORT subitem 13a? *

Copy and paste relevant sections from the manuscript (include quotes in quotation marks "like this" to indicate direct quotes from your manuscript), or elaborate on this item by providing additional information not in the ms, or briefly explain why the item is not applicable/relevant for your study

Yes: "During the course of the study, the public health authorities of Geneva requested that we create specific accesses for other institutions whose members were not part of the target population. A total of 652 accounts were created, out of which 569 (87.3%) were activated. After exclusion of accounts which had not completed the second questionnaire and of those belonging to institutions other than LTCFs, 295 answer sets were analyzed (Figure 9)." Figure 9 is the study flowchart.

13b) For each group, losses and exclusions after randomisation, together with reasons

Does your paper address CONSORT subitem 13b? (NOTE: Preferably, this is shown in a CONSORT flow diagram) *

Copy and paste relevant sections from the manuscript (include quotes in quotation marks "like this" to indicate direct quotes from your manuscript), or elaborate on this item by providing additional information not in the ms, or briefly explain why the item is not applicable/relevant for your study

Yes - this is fully detailed in Figure 9 (study flowchart)

13b-i) Attrition diagram

Strongly recommended: An attrition diagram (e.g., proportion of participants still logging in or using the intervention/comparator in each group plotted over time, similar to a survival curve) or other figures or tables demonstrating usage/dose/engagement.

	1	2	3	4	5	
subitem not at all important	○	○	○	○	◉	essential

Clear selection

Does your paper address subitem 13b-i?

Copy and paste relevant sections from the manuscript or cite the figure number if applicable (include quotes in quotation marks "like this" to indicate direct quotes from your manuscript), or elaborate on this item by providing additional information not in the ms, or briefly explain why the item is not applicable/relevant for your study

Yes - this is fully detailed in Figure 9 (study flowchart)

14a) Dates defining the periods of recruitment and follow-up

Does your paper address CONSORT subitem 14a? *

Copy and paste relevant sections from the manuscript (include quotes in quotation marks "like this" to indicate direct quotes from your manuscript), or elaborate on this item by providing additional information not in the ms, or briefly explain why the item is not applicable/relevant for your study

There was no follow-up as this was a "single-shot" intervention. The dates were already decided upon before the beginning of the study and are therefore mentioned in the METHODS ("[...] between November 5th and December 4th, 2020")

14a-i) Indicate if critical “secular events” fell into the study period

Indicate if critical “secular events” fell into the study period, e.g., significant changes in Internet resources available or “changes in computer hardware or Internet delivery resources”

	1	2	3	4	5	
subitem not at all important	○	○	○	○	◉	essential

Clear selection

Does your paper address subitem 14a-i?

Copy and paste relevant sections from the manuscript (include quotes in quotation marks "like this" to indicate direct quotes from your manuscript), or elaborate on this item by providing additional information not in the ms, or briefly explain why the item is not applicable/relevant for your study

Given the very limited timeframe, this was not detailed as there was no such event.

14b) Why the trial ended or was stopped (early)

Does your paper address CONSORT subitem 14b? *

Copy and paste relevant sections from the manuscript (include quotes in quotation marks "like this" to indicate direct quotes from your manuscript), or elaborate on this item by providing additional information not in the ms, or briefly explain why the item is not applicable/relevant for your study

Not applicable

15) A table showing baseline demographic and clinical characteristics for each group

NPT: When applicable, a description of care providers (case volume, qualification, expertise, etc.) and centers (volume) in each group

Does your paper address CONSORT subitem 15? *

Copy and paste relevant sections from the manuscript (include quotes in quotation marks "like this" to indicate direct quotes from your manuscript), or elaborate on this item by providing additional information not in the ms, or briefly explain why the item is not applicable/relevant for your study

Yes - Table 1 (Participant characteristics, from 36 long-term care facilities in Geneva, Switzerland.)

15-i) Report demographics associated with digital divide issues

In ehealth trials it is particularly important to report demographics associated with digital divide issues, such as age, education, gender, social-economic status, computer/Internet/ehealth literacy of the participants, if known.

	1	2	3	4	5	
subitem not at all important	○	○	○	○	◉	essential

Clear selection

Does your paper address subitem 15-i? *

Copy and paste relevant sections from the manuscript (include quotes in quotation marks "like this" to indicate direct quotes from your manuscript), or elaborate on this item by providing additional information not in the ms, or briefly explain why the item is not applicable/relevant for your study

Yes - these characteristics are given in Table 1, and the eHealth literacy issue mentioned in the discussion ("Lack of eHealth literacy can probably hardly be blamed for the lack of participation. Indeed, LTCF employees increasingly use digital devices in the course of their work, and recent surveys have shown that eHealth literacy was rather high in HCW.")

16) For each group, number of participants (denominator) included in each analysis and whether the analysis was by original assigned groups

16-i) Report multiple “denominators” and provide definitions

Report multiple “denominators” and provide definitions: Report N’s (and effect sizes) “across a range of study participation [and use] thresholds” [1], e.g., N exposed, N consented, N used more than x times, N used more than y weeks, N participants “used” the intervention/comparator at specific pre-defined time points of interest (in absolute and relative numbers per group). Always clearly define “use” of the intervention.

	1	2	3	4	5	
subitem not at all important	○	○	○	○	◉	essential

Clear selection

Does your paper address subitem 16-i? *

Copy and paste relevant sections from the manuscript (include quotes in quotation marks "like this" to indicate direct quotes from your manuscript), or elaborate on this item by providing additional information not in the ms, or briefly explain why the item is not applicable/relevant for your study

Yes, this is easily seen in Figure 9 (study flowchart) and mentioned in the text ("A total of 652 accounts were created, out of which 569 (87.3%) were activated. After exclusion of accounts which had not completed the second questionnaire and of those belonging to institutions other than LTCFs, 295 answer sets were analyzed (Figure 9).")

16-ii) Primary analysis should be intent-to-treat

Primary analysis should be intent-to-treat, secondary analyses could include comparing only “users”, with the appropriate caveats that this is no longer a randomized sample (see 18-i).

	1	2	3	4	5	
subitem not at all important	○	○	○	○	◉	essential

Clear selection

Does your paper address subitem 16-ii?

Copy and paste relevant sections from the manuscript (include quotes in quotation marks "like this" to indicate direct quotes from your manuscript), or elaborate on this item by providing additional information not in the ms, or briefly explain why the item is not applicable/relevant for your study

Yes, there was no other way this could have been performed given the study design

17a) For each primary and secondary outcome, results for each group, and the estimated effect size and its precision (such as 95% confidence interval)

Does your paper address CONSORT subitem 17a? *

Copy and paste relevant sections from the manuscript (include quotes in quotation marks "like this" to indicate direct quotes from your manuscript), or elaborate on this item by providing additional information not in the ms, or briefly explain why the item is not applicable/relevant for your study

Yes - this is all over the results section, but here is an example: "The willingness to change behavior was higher in the serious game group (82% [119/145], 95%CI 76% - 88% vs 56% [84/150], 95%CI 48% - 64%, P < .001), with an unadjusted odds ratio of 3.60 (95%CI 2.11 – 6.13, P < .001). After adjusting for professional category and baseline knowledge, using a random effects logistic regression model with LTCF as a random effect, the magnitude of the effect increased slightly with an odds ratio of 3.86 (95%CI 2.18 – 6.81, P < .001). The effect was not significantly affected by professional category (P = .46) or by baseline knowledge (P = .52). The ICC of 0.07 (95CI 0.01 - 0.33) suggests little correlation of responses within individual LTCFs, although the chi-bar-squared test showed that there was good evidence against the null hypothesis of no random effects (P = .046). A sensitivity analysis performed by excluding answers coming from LTCFs with <10 answers yielded an unadjusted odds ratio of 2.42 (95%CI 1.25 – 4.68, P = .009) and an adjusted odds ratio of 2.54 (95%CI 1.25 – 5.13, P = .01)."

17a-i) Presentation of process outcomes such as metrics of use and intensity of use

In addition to primary/secondary (clinical) outcomes, the presentation of process outcomes such as metrics of use and intensity of use (dose, exposure) and their operational definitions is critical. This does not only refer to metrics of attrition (13-b) (often a binary variable), but also to more continuous exposure metrics such as “average session length”. These must be accompanied by a technical description how a metric like a “session” is defined (e.g., timeout after idle time) [1] (report under item 6a).

	1	2	3	4	5	
subitem not at all important	○	○	○	○	◉	essential

Clear selection

Does your paper address subitem 17a-i?

Copy and paste relevant sections from the manuscript (include quotes in quotation marks "like this" to indicate direct quotes from your manuscript), or elaborate on this item by providing additional information not in the ms, or briefly explain why the item is not applicable/relevant for your study

Not applicable (single shot)

17b) For binary outcomes, presentation of both absolute and relative effect sizes is recommended

Does your paper address CONSORT subitem 17b? *

Copy and paste relevant sections from the manuscript (include quotes in quotation marks "like this" to indicate direct quotes from your manuscript), or elaborate on this item by providing additional information not in the ms, or briefly explain why the item is not applicable/relevant for your study

Yes - here is an example: "The willingness to change behavior was higher in the serious game group (82% [119/145], 95%CI 76% - 88% vs 56% [84/150], 95%CI 48% - 64%, P < .001), with an unadjusted odds ratio of 3.60 (95%CI 2.11 – 6.13, P < .001)."

18) Results of any other analyses performed, including subgroup analyses and adjusted analyses, distinguishing pre-specified from exploratory

Does your paper address CONSORT subitem 18? *

Copy and paste relevant sections from the manuscript (include quotes in quotation marks "like this" to indicate direct quotes from your manuscript), or elaborate on this item by providing additional information not in the ms, or briefly explain why the item is not applicable/relevant for your study

Yes: "A sensitivity analysis performed by excluding answers coming from LTCFs with <10 answers yielded an unadjusted odds ratio of 2.42 (95%CI 1.25 – 4.68, P = .009) and an adjusted odds ratio of 2.54 (95%CI 1.25 – 5.13, P = .01)." Prespecified analyses can easily be checked in our original protocol (https://www.researchprotocols.org/2020/12/e25595)

18-i) Subgroup analysis of comparing only users

A subgroup analysis of comparing only users is not uncommon in ehealth trials, but if done, it must be stressed that this is a self-selected sample and no longer an unbiased sample from a randomized trial (see 16-iii).

	1	2	3	4	5	
subitem not at all important	○	○	○	◉	○	essential

Clear selection

Does your paper address subitem 18-i?

Copy and paste relevant sections from the manuscript (include quotes in quotation marks "like this" to indicate direct quotes from your manuscript), or elaborate on this item by providing additional information not in the ms, or briefly explain why the item is not applicable/relevant for your study

Not applicable

19) All important harms or unintended effects in each group

(for specific guidance see CONSORT for harms)

Does your paper address CONSORT subitem 19? *

Copy and paste relevant sections from the manuscript (include quotes in quotation marks "like this" to indicate direct quotes from your manuscript), or elaborate on this item by providing additional information not in the ms, or briefly explain why the item is not applicable/relevant for your study

Not applicable

19-i) Include privacy breaches, technical problems

Include privacy breaches, technical problems. This does not only include physical "harm" to participants, but also incidents such as perceived or real privacy breaches [1], technical problems, and other unexpected/unintended incidents. "Unintended effects" also includes unintended positive effects [2].

	1	2	3	4	5	
subitem not at all important	○	○	○	○	◉	essential

Clear selection

Does your paper address subitem 19-i?

Copy and paste relevant sections from the manuscript (include quotes in quotation marks "like this" to indicate direct quotes from your manuscript), or elaborate on this item by providing additional information not in the ms, or briefly explain why the item is not applicable/relevant for your study

This was not explicitly mentioned, but there were no such problems during the course of this study. A continuous monitoring is in place on the main website (https://escape-covid19.ch/), the data of which are available at any time upon request to the last author.

19-ii) Include qualitative feedback from participants or observations from staff/researchers

Include qualitative feedback from participants or observations from staff/researchers, if available, on strengths and shortcomings of the application, especially if they point to unintended/unexpected effects or uses. This includes (if available) reasons for why people did or did not use the application as intended by the developers.

	1	2	3	4	5	
subitem not at all important	○	○	○	◉	○	essential

Clear selection

Does your paper address subitem 19-ii?

Copy and paste relevant sections from the manuscript (include quotes in quotation marks "like this" to indicate direct quotes from your manuscript), or elaborate on this item by providing additional information not in the ms, or briefly explain why the item is not applicable/relevant for your study

No qualitative feedback other than contained in the primary and secondary outcomes was gathered during this study

DISCUSSION

22) Interpretation consistent with results, balancing benefits and harms, and considering other relevant evidence

NPT: In addition, take into account the choice of the comparator, lack of or partial blinding, and unequal expertise of care providers or centers in each group

22-i) Restate study questions and summarize the answers suggested by the data, starting with primary outcomes and process outcomes (use)

Restate study questions and summarize the answers suggested by the data, starting with primary outcomes and process outcomes (use).

	1	2	3	4	5	
subitem not at all important	○	○	○	○	◉	essential

Clear selection

Does your paper address subitem 22-i? *

Copy and paste relevant sections from the manuscript (include quotes in quotation marks "like this" to indicate direct quotes from your manuscript), or elaborate on this item by providing additional information not in the ms, or briefly explain why the item is not applicable/relevant for your study

Yes: "After following an online learning path, most participants reported that they were willing to change their IPC behavior. The serious game « Escape COVID-19 » was, however, significantly more successful at inducing that change than the simple presentation of IPC guidelines." and "Factors underlying the willingness to change IPC behavior were very similar between groups, with the feeling of playing an important role against the epidemic being most prominent, superseding even the information given in the training material"

22-ii) Highlight unanswered new questions, suggest future research

Highlight unanswered new questions, suggest future research.

	1	2	3	4	5	
subitem not at all important	○	○	○	○	◉	essential

Clear selection

Does your paper address subitem 22-ii?

Copy and paste relevant sections from the manuscript (include quotes in quotation marks "like this" to indicate direct quotes from your manuscript), or elaborate on this item by providing additional information not in the ms, or briefly explain why the item is not applicable/relevant for your study

Yes: "This serious game might not be equally effective in all populations, and IPC messages might differ from one region to another. By virtue of its flexible design, “Escape COVID-19” can be updated rather easily and should now be tested on other populations. It has been fully translated in English and is in the process of being translated in German and Italian to allow its deployment at the Swiss national level.
"

20) Trial limitations, addressing sources of potential bias, imprecision, and, if relevant, multiplicity of analyses

20-i) Typical limitations in ehealth trials

Typical limitations in ehealth trials: Participants in ehealth trials are rarely blinded. Ehealth trials often look at a multiplicity of outcomes, increasing risk for a Type I error. Discuss biases due to non-use of the intervention/usability issues, biases through informed consent procedures, unexpected events.

	1	2	3	4	5	
subitem not at all important	○	○	○	○	●	essential

Clear selection

Does your paper address subitem 20-i? *

Copy and paste relevant sections from the manuscript (include quotes in quotation marks "like this" to indicate direct quotes from your manuscript), or elaborate on this item by providing additional information not in the ms, or briefly explain why the item is not applicable/relevant for your study

Yes: "This study has several limitations. First, even though the probability of executing an action is strongly linked to the intention of performing it, one can hardly be certain that LTCF employees claiming they are willing to change their IPC behavior will actually change it. Field observations would be necessary to ascertain this aspect, along with a different study design as both groups ultimately accessed the serious game in the present study. Another important limitation is that we did not reach the sample size we had expected. Indeed, while we had hoped for at least 800 participants, the actual number of accounts activated by LTCF employees was rather lower. This low figure raises many questions and hypotheses. Indeed, while at least a complete answer set was obtained for each individual LTCF, less than 10 complete answer sets were given by more than three quarters (28/36) of all LTCFs under the jurisdiction of the public health authorities of Geneva, with almost half of those (12/36) giving less than 5 complete answer sets. It might therefore be assumed that, while all LTCF managers received the information regarding the study, many decided not to forward it to their employees. This was an unexpected finding; however, this study was not designed to assess the reasons underlying this decision. Hypotheses can however be drawn, some of which are more concerning than others. Among the least concerning, fear of overloading already overworked LTCF employees with information, an insufficient number of reminders, or the simple lack of regularly updated mailing lists could partly explain the low participation rate witnessed in many LTCFs. Moreover, some managers might have felt that the material, which originated from a university hospital, was not in line with their situation. This hypothesis is however challenged by the fact that this impression, though asked for, was reported by 10 participants only. Lack of eHealth literacy can probably hardly be blamed for the lack of participation. Indeed, LTCF employees increasingly use digital devices in the course of their work, and recent surveys have shown that eHealth literacy was rather high in HCW. Among the most disturbing hypotheses, a low level of concern of some LTCF managers, a potential mistrust in health authorities, in the institution authoring the game, or even in IPC guidelines, cannot be ruled out. The will to avoid spreading information that could lead to an increase in the use of PPE items, and, therefore, to an increase in material costs, seems however unlikely. Regardless of the reason, the ability of health authorities to successfully convey critical messages by efficient vectors to LTCF employees should be assessed specifically to solve potential communication issues. The creation of IPC focal points in each LTCF is a path that could be explored."

21) Generalisability (external validity, applicability) of the trial findings

NPT: External validity of the trial findings according to the intervention, comparators, patients, and care providers or centers involved in the trial

21-i) Generalizability to other populations

Generalizability to other populations: In particular, discuss generalizability to a general Internet population, outside of a RCT setting, and general patient population, including applicability of the study results for other organizations

	1	2	3	4	5	
subitem not at all important	○	○	○	○	◉	essential

Clear selection

Does your paper address subitem 21-i?

Copy and paste relevant sections from the manuscript (include quotes in quotation marks "like this" to indicate direct quotes from your manuscript), or elaborate on this item by providing additional information not in the ms, or briefly explain why the item is not applicable/relevant for your study

Yes: "This serious game might not be equally effective in all populations, and IPC messages might differ from one region to another. By virtue of its flexible design, “Escape COVID-19” can be updated rather easily and should now be tested on other populations. It has been fully translated in English and is in the process of being translated in German and Italian to allow its deployment at the Swiss national level. To enhance its visibility, publicizing actions similar to those used to promote another recently developed serious game (“COVID-19 – Did You Know?) should be considered. Currently, “Escape COVID-19” is freely available to play online, and the corresponding author can be contacted at any time to obtain a SCORM package in either French or English, pending translation in other languages."

21-ii) Discuss if there were elements in the RCT that would be different in a routine application setting

Discuss if there were elements in the RCT that would be different in a routine application setting (e.g., prompts/reminders, more human involvement, training sessions or other co-interventions) and what impact the omission of these elements could have on use, adoption, or outcomes if the intervention is applied outside of a RCT setting.

	1	2	3	4	5	
subitem not at all important	○	○	○	○	◉	essential

Clear selection

Does your paper address subitem 21-ii?

Copy and paste relevant sections from the manuscript (include quotes in quotation marks "like this" to indicate direct quotes from your manuscript), or elaborate on this item by providing additional information not in the ms, or briefly explain why the item is not applicable/relevant for your study

This is implicit: "It has been fully translated in English and is in the process of being translated in German and Italian to allow its deployment at the Swiss national level. To enhance its visibility, publicizing actions similar to those used to promote another recently developed serious game ("COVID-19 – Did You Know?) should be considered."

OTHER INFORMATION

23) Registration number and name of trial registry

Does your paper address CONSORT subitem 23? *

Copy and paste relevant sections from the manuscript (include quotes in quotation marks "like this" to indicate direct quotes from your manuscript), or elaborate on this item by providing additional information not in the ms, or briefly explain why the item is not applicable/relevant for your study

Yes - our protocol was published and the IRRID: DERR1-10.2196/25595 automatically attributed thanks to JRP

24) Where the full trial protocol can be accessed, if available

Does your paper address CONSORT subitem 24? *

Cite a Multimedia Appendix, other reference, or copy and paste relevant sections from the manuscript (include quotes in quotation marks "like this" to indicate direct quotes from your manuscript), or elaborate on this item by providing additional information not in the ms, or briefly explain why the item is not applicable/relevant for your study

Yes: https://www.researchprotocols.org/2020/12/e25595

25) Sources of funding and other support (such as supply of drugs), role of funders

Does your paper address CONSORT subitem 25? *

Copy and paste relevant sections from the manuscript (include quotes in quotation marks "like this" to indicate direct quotes from your manuscript), or elaborate on this item by providing additional information not in the ms, or briefly explain why the item is not applicable/relevant for your study

Yes: "Funding: None"

X27) Conflicts of Interest (not a CONSORT item)

X27-i) State the relation of the study team towards the system being evaluated

In addition to the usual declaration of interests (financial or otherwise), also state the relation of the study team towards the system being evaluated, i.e., state if the authors/evaluators are distinct from or identical with the developers/sponsors of the intervention.

	1	2	3	4	5	
subitem not at all important	○	○	○	○	◉	essential

Clear selection

Does your paper address subitem X27-i?

Copy and paste relevant sections from the manuscript (include quotes in quotation marks "like this" to indicate direct quotes from your manuscript), or elaborate on this item by providing additional information not in the ms, or briefly explain why the item is not applicable/relevant for your study

Yes: "This investigator was not part of the serious game development team and did not co-author the original publication as we wanted to avoid any potential conflict of interest." and "we developed a serious game called “Escape COVID-19”"

About the CONSORT EHEALTH checklist

As a result of using this checklist, did you make changes in your manuscript? *

- ○ yes, major changes
- ○ yes, minor changes
- ◉ no

What were the most important changes you made as a result of using this checklist?

Not applicable - we had already used the checklist to design our protocol and manuscript

How much time did you spend on going through the checklist INCLUDING making changes in your manuscript *

120 minutes

As a result of using this checklist, do you think your manuscript has improved? *

- (x) yes
- ( ) no
- ( ) Other:

Would you like to become involved in the CONSORT EHEALTH group?

This would involve for example becoming involved in participating in a workshop and writing an "Explanation and Elaboration" document

- (x) yes
- ( ) no
- ( ) Other:

Clear selection

Any other comments or questions on CONSORT EHEALTH

We find this checklist so useful that we use it to design our protocols (and manuscripts)

STOP - Save this form as PDF before you click submit

To generate a record that you filled in this form, we recommend to generate a PDF of this page (on a Mac, simply select "print" and then select "print as PDF") before you submit it.

When you submit your (revised) paper to JMIR, please upload the PDF as supplementary file.

Don't worry if some text in the textboxes is cut off, as we still have the complete information in our database. Thank you!

Final step: Click submit !

Click submit so we have your answers in our database!

Submit

Never submit passwords through Google Forms.

This content is neither created nor endorsed by Google. Report Abuse - Terms of Service - Privacy Policy

Google Forms
